# Assessing biological factors affecting postspeciation introgression

**DOI:** 10.1002/evl3.159

**Published:** 2020-02-28

**Authors:** Jennafer A. P. Hamlin, Mark S. Hibbins, Leonie C. Moyle

**Affiliations:** ^1^ Department of Plant Biology University of Georgia Athens Georgia 30602; ^2^ Department of Biology Indiana University Bloomington Indiana 47405

**Keywords:** Geographic proximity, hybridization, mating system variation, *Solanum*, tomato, whole‐genome

## Abstract

An increasing number of phylogenomic studies have documented a clear “footprint” of postspeciation introgression among closely related species. Nonetheless, systematic genome‐wide studies of factors that determine the likelihood of introgression remain rare. Here, we propose an a priori hypothesis‐testing framework that uses introgression statistics—including a new metric of estimated introgression, *D*_p_—to evaluate general patterns of introgression prevalence and direction across multiple closely related species. We demonstrate this approach using whole genome sequences from 32 lineages in 11 wild tomato species to assess the effect of three factors on introgression—genetic relatedness, geographical proximity, and mating system differences—based on multiple trios within the “ABBA–BABA” test. Our analyses suggest each factor affects the prevalence of introgression, although our power to detect these is limited by the number of comparisons currently available. We find that of 14 species pairs with geographically “proximate” versus “distant” population comparisons, 13 showed evidence of introgression; in 10 of these cases, this was more prevalent between geographically closer populations. We also find modest evidence that introgression declines with increasing genetic divergence between lineages, is more prevalent between lineages that share the same mating system, and—when it does occur between mating systems—tends to involve gene flow from more inbreeding to more outbreeding lineages. Although our analysis indicates that recent postspeciation introgression is frequent in this group—detected in 15 of 17 tested trios—estimated levels of genetic exchange are modest (0.2–2.5% of the genome), so the relative importance of hybridization in shaping the evolutionary trajectories of these species could be limited. Regardless, similar clade‐wide analyses of genomic introgression would be valuable for disentangling the major ecological, reproductive, and historical determinants of postspeciation gene flow, and for assessing the relative contribution of introgression as a source of genetic variation.

**Impact Statement**

The formation of new species is traditionally viewed as a tree‐like branching process, in which species are discrete branches that no longer share an ongoing genetic connection with other, equally discrete, species. Recently, this view has been challenged by numerous studies examining genealogical patterns across entire genomes (all the DNA of an organism); these studies suggest that the exchange of genetic variants between different species (known as “introgression”) is much more common than previously appreciated. This unexpected observation raises questions about which conditions are most important in determining whether species continue to exchange genes after they diverge. Factors such as physical proximity, differences in reproductive mechanisms, and time since species shared a common ancestor might all contribute to determining the prevalence of introgression. But to evaluate the general importance of these factors requires more than individual cases; many species comparisons, which vary systematically in one or more of these conditions, are needed. Here, we use whole‐genome information from 32 lineages to evaluate patterns of introgression among multiple species in a single, closely related group—the wild tomatoes of South America. We contrast these patterns among pairs of lineages that differ in their geographical proximity, reproductive system, and time since common ancestry to assess the individual influence of each condition on the prevalence of introgression. In this case, we find some evidence that the prevalence or direction of introgression is associated with each of these effects. By systematically assessing the influence of general ecological and evolutionary conditions on the frequency of postspeciation introgression, our study provides a straightforward, generalizable, hypothesis‐testing framework for similar analyses of introgression in other groups in the future.

The prevalence of hybridization among species and the importance of introgression for shaping species evolution are historically contentious questions (Mallet 2005, 2008). Although traditionally viewed to be more common among plants (Anderson and de Winton 1931; Stebbins 1970), evidence of hybridization and introgression is emerging for an increasingly broad range of organisms (Mallet et al. 2016). Perhaps the most famous contemporary example involves Neanderthal and modern human lineages, in which approximately 1–4% of Neanderthal genome is inferred to have introgressed into some human populations (Green et al. 2010). Quantifying the frequency and amount of introgression is important for understanding the historical dynamics of closely related lineages, as well as the potential sources of genetic variation that could fuel ongoing evolutionary change. For example, if sufficiently common, gene flow among species could act as a significant source of adaptive alleles, as has been observed for mimicry pattern alleles in *Heliconius* butterflies. Adaptive introgression is likely to be more prevalent among recently diverged lineages, where the accumulation of hybrid incompatibilities is not so advanced that it prevents the exchange of unconditionally adaptive loci when lineages come into contact. Nonetheless, the clade‐wide prevalence of introgression events, and therefore their relative importance in shaping the evolutionary trajectory of close relatives, is only now beginning to be assessed (Folk et al. 2018).

From a genomic perspective, introgression leaves a detectable “footprint”: introgressed regions show distinctive patterns of historical relatedness that differ from nonintrogressed regions, because they are most closely related to the donor species rather than the recipient genome in which they are found (Payseur and Rieseberg 2016). Accordingly, genome‐wide data are ideal for characterizing the prevalence of hybridization because the data provide a detailed picture of phylogenetic relationships at loci across the genome, including in genomic regions that show patterns of relatedness inconsistent with the species as a whole. Beyond the human and butterfly examples, genome‐wide data have been used to infer past introgression events among species in groups as diverse as *Saccharomyces* yeast (Morales and Dujon 2012), *Anopheles* mosquitoes (Fontaine et al. 2015), wild tomatoes (Pease et al. 2016), and *Drosophila* (Turissini and Matute 2017). Although revealing the extent and timing of gene flow events is interesting in individual cases, there are few tests of the generality of introgression across whole groups of closely related species, including whether it systematically varies in frequency or extent under different biological conditions.

Some of the factors that could influence the frequency of hybridization and subsequent introgression include phylogenetic relatedness (i.e., genetic distance), geographical proximity, and biological factors that affect the likelihood and direction of reproductive events, such as differences in mating system. In the first case, because the strength of reproductive isolation is expected to accumulate with the amount of time since lineages diverged (Coyne and Orr 1989), more genetic exchange might be expected to occur between more closely related species, with diminishing rates accompanying increasing lineage differentiation. Second, genetic exchange is more likely to occur among species in close geographic proximity, where they can potentially come into physical and therefore reproductive contact (Harrison 2012). Determining the level of spatial proximity that allows gene exchange can be challenging, as it likely depends upon numerous biological factors (e.g., dispersal mechanisms) and abiotic factors (e.g., physical barriers to dispersal). Nonetheless, a reasonable expectation is that hybridization is more likely with closer physical proximity compared with greater physical distance among lineages. For example, numerous hybrid zone studies demonstrate that the proportion of individuals with mixed ancestry usually decreases with geographic distance from the hybrid zone (Harrison and Larson 2016, 2014).

Third, factors that specifically influence the timing and success of reproductive events are also expected to influence the likelihood of hybridization and introgression. For example, mating system variation (such as outcrossing vs. inbreeding, or self‐incompatible vs. self‐compatible) can influence introgression, either immediately by affecting the likelihood of successful mating between species or in the longer term by influencing the likelihood that introgressed loci will persist in the recipient lineage. In the first instance, mating system differences can cause predictable asymmetries in the success of initial crosses among species. This can occur either via differences in the size or shape of reproductive organs that can lead to asymmetric mechanical isolation among lineages (e.g., where outcrossing species can fertilize inbreeding species, but not vice versa; Levin 1978; Brothers and Delph 2017) or—especially in plants—via differences in the presence/absence of genetically determined self‐incompatibility systems, whereby pollen from self‐incompatible species can fertilize ovules of self‐compatible species, but self‐incompatible plants actively reject pollen from self‐compatible species (e.g., in *Nicotiana*: Anderson and de Winton 1931; *Petunia*: Mather and Edwardes 1943; and *Solanum*: McGuire and Rick 1954). In both mechanical and active‐rejection cases, outcrossing species are more likely to donate alleles to more inbreeding species compared to the reciprocal direction of gene flow, reducing the potential for gene flow specifically between species with unalike mating systems. Similarly, the longer term likelihood that introgressed loci will persist in recipient lineages could vary based on the mating system of the donor and recipient lineages—especially due to the strong effects that mating system can have on relative rates of inbreeding. This is because mutational load and the efficacy of selection are expected to differ between species with histories of more or less inbreeding and different effective population sizes (*N_e_*) (Lande and Schemske 1985; Charlesworth et al. 1990; Busch 2005; Harris and Nielsen 2016; Juric et al. 2016). For example, introgression from outbreeding to inbreeding populations could be especially disfavored both because donor alleles are expected to have stronger deleterious fitness effects (due to genetic load that can persist in outbreeders) and because the smaller *N_e_* recipient population is less effective at disassociating these from other nondeleterious loci before they are purged (Ruhsam et al. 2011; Brandvain et al. 2014). In comparison, the exchange of alleles between lineages with similar mating systems (therefore levels of outcrossing and/or *N_e_*) should be less constrained by these factors. In general, then, no matter whether affected by initial crossing differences (from mechanical or active rejection asymmetries) or differences in the historical factors determining genetic load and effective population size, gene flow between lineages that differ in their mating system might be expected to be more constrained than gene flow between lineages with similar mating systems.

Although these factors are expected to influence the rate and likelihood of gene flow between species, there are few systematic tests of their general importance in shaping the prevalence of postspeciation introgression. Here, our goal is to use whole genome data to systematically evaluate several of these effects on genome‐wide patterns of postspeciation introgression across a closely related clade of species. To assess introgression, we use the “ABBA–BABA” test (also known as the *D*‐statistic; Green et al. 2010; Durand et al. 2011). This test detects introgression by comparing the frequency of alternate ancestral (“A”) and derived (“B”) allele patterns among four taxa, where the species tree has the allele pattern BBAA (Fig. 1). In the absence of gene flow, the alternate minority patterns of ABBA and BABA should be approximately equally frequent, as they have an equal chance of either coalescence pattern under incomplete lineage sorting (ILS; Durand et al. 2011). In comparison, an excess of ABBA patterns indicates gene flow between lineage P2 and P3, and excess BABA indicates gene flow between lineage P1 and P3 (Fig. 1).

**Figure 1:**
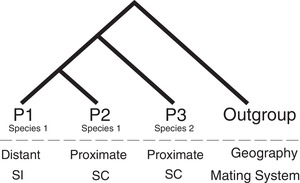
Structured ABBA–BABA tests to evaluate a priori hypotheses about the expected prevalence of introgression due to geographical proximity and/or lineage differences in mating system. For example, if introgression occurs more frequently between geographically closer accessions, more minority topologies should support a closer relationship between P2 and P3, compared to P1 and P3, and the genome‐wide mean *D*‐statistic is expected to be positive.

Importantly, the structure of the ABBA–BABA test allows us to test a priori hypotheses about the expected prevalence of introgression in multiple parallel comparisons. In particular, we can intentionally construct tests of a specific introgression hypothesis by consistently positioning taxa in the P1, P2, and P3 positions in a four‐taxon tree, so that P2 and P3 are always predicted to experience more introgression than P1 and P3 (Fig. 1). For example, if geographic proximity per se affects the amount of introgression between two species, in a case where P1 and P2 are conspecific populations but P2 is more geographically proximate to populations of a second species (P3), then our a priori expectation is that elevated introgression will be observed as an excess of ABBA (i.e., evidence of gene flow between P2 and P3) rather than BABA (gene flow between P1 and P3; Fig. 1). Multiple different four‐taxon tests with the same structure can then be used to evaluate whether geographic proximity is more frequently associated with evidence of postspeciation introgression. A similar structure can be used to test the a priori expectation that gene flow is expected to be more frequent between lineages with shared versus different mating systems. Within trios that show evidence for gene flow, the prevalent direction of gene flow between different mating systems can be further assessed with additional statistics that evaluate the direction of introgression (e.g., Hibbins and Hahn 2019). More generally, four‐taxon tests that involve increasing evolutionary divergence between the P1/P2 and P3 lineages can be used to evaluate evidence that introgression is on average more prevalent between more closely related taxa.

Here, we use this a priori hypothesis‐testing framework to assess the prevalence and frequency of introgression among wild tomato lineages (*Solanum* section *Lycopersicum*) depending upon (a) geographical proximity, (b) differences in mating system, and (c) evolutionary distance. The wild tomato group consists of 12 closely related (<2.5 million years ago) and rapidly diverging wild species (Peralta et al. 2008; Rodriguez et al. 2009; Pease et al. 2016). Species ranges occur across diverse latitudinal, altitudinal, and environmental gradients in Andean northwestern South America, and on the Galapagos Islands (Moyle 2008; Haak et al. 2014; Figure 2). Based on geographical records from thousands of field collections, species vary in their preferred environmental habitats, but at least eight different species pairs are sympatric in some part of their natural range (Moyle 2008; Nakazato et al. 2010). Lineages (species, and some populations within species) also vary in their functional outcrossing rates, primarily due to the presence/absence of genetically determined self‐incompatibility (SI); although the effective rate of outcrossing does vary among lineages that are genetically self‐compatible (SC), SI lineages always show evidence of greater outcrossing compared to SC lineages (Bedinger et al. 2011; Vosters et al. 2014; Pease et al. 2016). Moreover, prior evidence of introgression events between specific lineages (e.g., Pease et al. 2016, Beddows et al. 2017) and the ability to generate F1 and later‐generation hybrids in the greenhouse (e.g., Moyle 2008; Rick 1979) indicate the possibility that introgression could shape genomes in this group. Using whole‐genome data from 32 closely related accessions across 11 species of wild tomato (Fig. 2), our goal here was to systematically test hypotheses about the prevalence of introgression to make general inferences about the role and importance of particular factors in the frequency of cross‐species hybridization, and to begin to assess the potential importance of introgression in shaping genome content and evolutionary trajectories in this clade.

**Figure 2:**
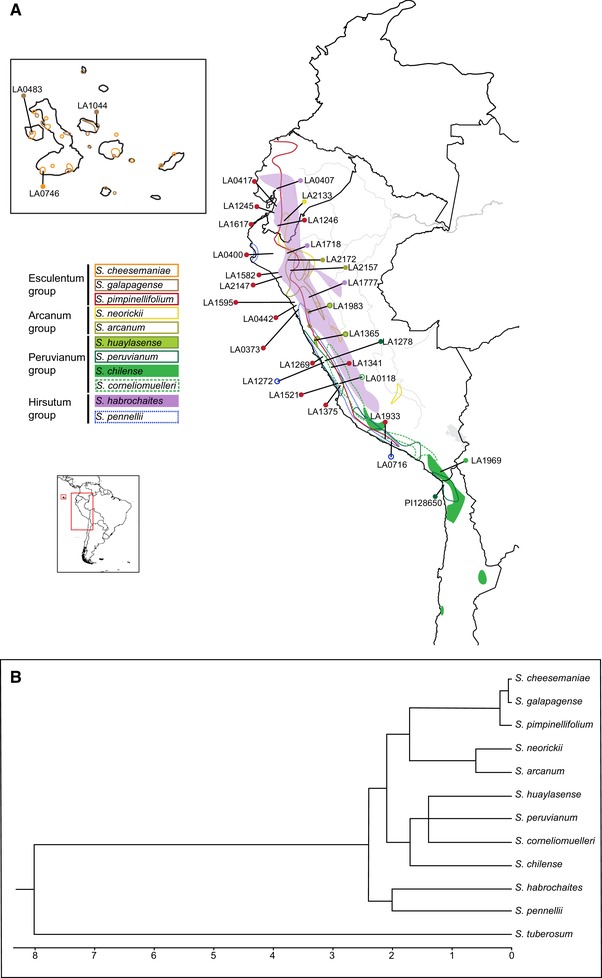
(A) Natural ranges of 11 species of *Solanum* used in this analysis, and the geographical locations of the specific accessions used in this study. (B) Phylogenetic relationships for the species used in this study along with the estimated times of divergence in millions of years based on Pease et al. 2016.

## Methods

### SEQUENCING DATA AND MAPPING TO REFERENCE GENOME

Our analyses used data from three published whole genome‐sequencing projects (Aflitos et al. 2014; Lin et al. 2014; Hardigan et al. 2016). Specifically, we obtained raw reads either as fastq or SRA files for genomes of 32 wild *Solanum* individuals from the tomato clade (*Solanum* section *Lycopersicum*), each from a different accession (historical population collection), along with *Solanum tuberosum* (potato; Hardigan et al. 2016), which we used as the outgroup in all comparisons (Table S1). Original population collections were made between approximately 40 and 60 years ago and maintained in germplasm collections; each accession is represented by a single sequenced individual, and number of accessions (genomes) per species ranged from one to 15. The availability of >1 accession for some of these species enabled us to contrast two conspecific accessions (as P1 and P2) within each of our trios. To combine data from the different sequencing projects, we trimmed and remapped raw reads back to the reference genome of domesticated tomato, *S. lycopersicum* version 2.50 (The Tomato Genome Consortium 2012), using standard practices for mapping and quality‐filtering (Text in the Supporting Information).

### HYPOTHESIS TESTING WITH THE *D*‐STATISTIC

We used the ABBA–BABA test to assess evidence for the presence of gene flow in a set of four‐taxon tests. The results of each ABBA–BABA test can be expressed in terms of Patterson's *D*‐statistic, calculated as (#ABBA – #BABA)/(#ABBA + #BABA) for all biallelic sites in the multiple sequence alignment (Green et al. 2010; Durand et al. 2011). The *D*‐statistic therefore summarizes both evidence for introgression and the specific pair of taxa that are differentially exchanging alleles; positive values of *D* indicate P2 and P3 are exchanging more alleles (an excess of ABBA) and negative values indicate more gene exchange between P1 and P3 (an excess of BABA). In the analyses performed here, all our four‐taxon tests used conspecific accessions for P1 and P2, therefore these tests only evaluate evidence for very recent differential introgression with a second species (P3)—that is, events that have occurred since P1 and P2 shared a most recent common ancestor within the same species. We used multiple replicate four‐taxon tests to evaluate three a priori expectations:

(1) Postspeciation introgression is more prevalent between geographically closer versus more distant lineages. Four‐taxon tests were structured so that the P2 accession was spatially closer to the P3 species while the P1 accession was more distant (Fig. 1). In this case, we expect a systematic excess of positive values of the resulting *D*‐statistics. Species comparisons and specific P1, P2, and P3 accessions were identified for these tests based on known species ranges and geographical locations of the sequenced accessions (see Text in the Supporting Information for our specific criteria). Because these analyses are constrained by the available sequenced genotypes, the actual geographic distances involved vary broadly among four‐taxon tests (Table S2), so that this analysis is an imperfect reflection of close spatial proximity; however, the structure of each test means we are still able to systematically compare the effect of greater (“proximate”) versus less (“distant”) geographic proximity between two species (P1/P2, and P3) on detected patterns of introgression.
(2) Postspeciation introgression is more prevalent among lineages that share mating system. Here, four‐taxon tests were structured so that the P2 accession had an inferred mating system that matched the P3 lineage, whereas the P1 accession mating system differed from the P3 lineage. For example, where P1 was self‐incompatible (SI) and P2 was self‐compatible (SC), P3 was an SC accession of a different species. With this structure, we also expect a systematic excess of positive *D*‐statistics. Within our dataset, there are only three species for which we had whole genome sequence data from both SI and SC accessions—*S. arcanum*, *S. habrochaites*, and *S. peruvianum—*that could therefore serve as the P1/P2 species in these tests.
(3) Postspeciation introgression is more prevalent between lineages that are more closely related. In this case, we expect that the estimated magnitude of introgression should decrease as evolutionary (genetic) distance between (P1, P2) and P3 increases within the four‐taxon test. We calculated pairwise genetic distance for each comparison by taking the average genetic distance for the two comparisons within the focal trio (i.e., P1 with P3 and P2 with P3), based on genome‐wide site differences between accessions. To estimate the magnitude of introgression for each trio, we generated an index for the proportion of genome introgressed—*D*_p_—based on site counts used in ABBA–BABA test, as described below. In this test, *D*_p_ is expected to be negatively associated with genetic distance, across all our four‐taxon combinations.

The supplemental text provides a more detailed description of how each of these factors (geographical proximity, mating system, and genetic distance) was defined or determined for individual four‐taxon tests. Note also that using common trios in both geographic and mating system tests could introduce bias into our results if these two factors are consistently associated with each other, such that the relative geographical proximity of P1 and P2 accessions to a P3 lineage consistently covaried with the relative mating system identity of P1 and P2 with respect to the mating system of the P3 lineage. This is not the case in our data, where there is no consistent relationship between these factors, including for the two trios that were used in both tests (see Results); indeed, most trios do not meet the criteria for testing both factors, and therefore could not be used to assess both geography and mating system effects (Results).

### CALCULATING *D*‐STATISTICS

To estimate *D*‐statistics for each trio, we first generated a multiple sequence alignment consisting of 99,302,292 variable sites across the 32 tomato accessions, plus the potato outgroup, using MVFtools version 0.5.4 (Pease and Rosenzweig 2018). Empirical estimation of the mean genome‐wide D‐statistics, in addition to block bootstrapping to evaluate significance, was done using a custom python script, which is available at https://doi.org/10.5061/dryad.tb2rbnzwj. For each bootstrap replicate, 1000 windows of 100 kb each were sampled with replacement from the empirical alignment and concatenated to generate a bootstrapped alignment 100 mb in size. For each trio, 1000 bootstrap replicates were performed to generate a distribution of *D*‐statistics. Standard deviation, standard error, and 95% confidence intervals of *D* for each trio were estimated using this distribution. Finally, to estimate *P*‐values, we asked how often the bootstrap distribution of the *D*‐statistics overlapped 0; so, for an empirical *D* value > 0, this would be the number of replicates where *D* ≤ 0, and vice versa. *P*‐values were adjusted for multiple tests (17 trios). In addition to mean genome‐wide *D* for four‐taxon test, to look at more fine‐grained patterns of *D*, we also examined estimates of *D* within individual 100‐kb windows across the genome. These analyses included only windows with >20 single nucleotide polymorphisms (SNPs; Table S3) so as to exclude those windows low power to accurately estimate *D*. Here, a 100‐kb window was considered a *D* outlier when a *z*‐score calculated for that window suggested that it deviated from other windows (the absolute value of mean *D* divided by the standard deviation, i.e., abs(mean*D*)/sd*D*) as in Pease et al. 2016).

### ESTIMATING THE ADMIXTURE PROPORTION (*D*_p_)

To generate estimates of the net proportion of the genome originating from a history of introgression in each trio, we used an adjusted version of the *D*‐statistic:

$$\;D_{p}=\;\frac{|\mathrm{ABBA}-\mathrm{BABA}|}{\left(\mathrm{ABBA}+\mathrm{BABA}+\mathrm{BBAA}\right)}.$$

As in the standard *D*‐statistic, BBAA is the expected allelic pattern when the observed allelic variation follows the topology of relationships described in the species tree (i.e., (((P1, P2), P3), P4)), where the “A” allele indicates the ancestral state at this site. *D*_p_ therefore adjusts the difference between ABBA and BABA counts so that they are a proportion of the total number of variable sites (ABBA + BABA + BBAA). We estimated *D*_p_ for each trio from our generated multiple sequence alignment, using a python script available at https://doi.org/10.5061/dryad.tb2rbnzwj. Note that because this statistic provides a simple global estimate of the net fraction of the genome that has been introgressed for each trio, it can underestimate total introgression if there has been gene flow both between P1 and P3, and between P2 and P3.

To more directly evaluate the performance of *D*_p_ as an estimate of the proportion of introgressed genome, we simulated multi‐locus alignments using the coalescent simulator *ms* (Hudson 2002) and the sequence simulator Seq‐Gen (Rambaut and Grass 1997). Each simulation consisted of 10,000 non‐recombining loci with three ingroup taxa and an outgroup (P4), and the species tree topology (((P1, P2), P3), P4). P1 and P2 split at a time of 1.2N generations; P3 from P1/P2 at 2.4N generations; and the outgroup at 16N generations. Introgression was specified at one of two times (0.2N generations or 0.04N generations), and in one of two directions (P2 → P3 or P3 → P2). For each combination of direction and timing, 100 replicate simulations were performed for each of 10 different values of the admixture proportion, ranging from 0.05 to 0.95. Simulated gene trees were passed to Seq‐Gen, and 10‐kb alignments were simulated from each locus under the Jukes‐Cantor model with θ = 0.001. Further details on the choice of parameters are provided in Methods in the Supporting Information.

The resulting datasets, each consisting of 10,000 loci of 10 kb each, were each concatenated to estimate *D* and *D*_p_ using a python script available at https://doi.org/10.5061/dryad.tb2rbnzwj. We evaluate the performance of both metrics by comparing their estimates to what would be expected from a “perfect” estimator of the admixture proportion.

### DETERMINING THE PRIMARY DIRECTION OF INTROGRESSION

For all trios in which we inferred significant introgression, we also determined the primary direction of introgression using the *D*_2_ statistic (Hibbins and Hahn 2019). Briefly, this statistic is based on a prediction of the multispecies network coalescent that, in a rooted three‐taxon tree, the direction of introgression between one nonsister pair can affect the degree of divergence between the nonsister pair not involved in introgression. For a three‐taxon tree with the species topology ((A, B), C), it is defined as follows:

$$D_{2}=\left(dAC|\mathrm{AB}\right)-\left(dAC|\mathrm{BC}\right)\;,$$

where *dAC* is the genetic distance between the nonsister pair that is uninvolved in introgression. It is measured conditionally on two different gene tree topologies; the tree concordant with the species branching order (“AB”) and the tree concordant with the inferred history of introgression (“BC”). The statistic is the genome‐wide difference in *AC* divergence between these two topologies. Assuming a constant *N_e_* and an admixture proportion of 0.5, the value of *D*_2_ is expected to be 0 for primarily P3 → P2 introgression, and it is expected to be positive when introgression is primarily P2 → P3. However, lineage‐specific variation in *N_e_* and deviations of the admixture proportion from 50% can both cause nonzero *D*_2_ values that are unrelated to the direction of introgression. Both these assumptions are violated in our study system, which means hypothesis testing cannot be done under the null hypothesis of *D*_2_ = 0. Instead, we simulated a distribution of *D*_2_ statistics for each trio under the null hypothesis of P3 → P2 introgression, using *ms* and Seq‐Gen, and evaluated the deviation of observed values from this null. We used the empirical estimates of the admixture proportion (*D*_p_) obtained for each trio, so deviations from 50% are incorporated explicitly into the null distribution for each test.

To estimate split times for each trio, we used species‐level estimates from the molecular clock phylogeny in Pease et al. (2016), a phylotranscriptomic study of 29 accessions from across all 12 wild tomato species as well as several outgroups. For P1/P2 splits where multiple accessions for a given species were unavailable in Pease et al. (2016), we used the average within‐species split time for the other species in the same subclade as a proxy. We used estimates of heterozygosity from Pease et al. (2016) as proxies for lineage‐specific variation in theta or the population mutation rate. *D*_2_ is largely robust to variation in theta between internal branches at introgressed versus nonintrogressed loci (at least up to twofold differences), but does have some sensitivity to variation in the split times when introgression is primarily P2 → P3 (Hibbins and Hahn 2019). Other deviations in theta, including between the ancestral population or tip branches, should not bias the statistic, as they affect introgressed and nonintrogressed loci equally. Branch lengths were converted from units of years to coalescent units using values of *N_e_* = 1.0 × 10^6^ and a generation time of one generation every two years. We simulated 100‐kb non‐recombining loci, with the number of loci corresponding to the number of 100‐kb windows inferred in the empirical alignment. A total of 1000 replicate simulations were performed for each trio, and *P*‐values were estimated by calculating the proportion of null *D*_2_ values at least as extreme as the observed value of *D*_2_ for that trio. Scripts for performing this analysis are available at https://doi.org/10.5061/dryad.tb2rbnzwj.

### ANALYZING *D*‐STATISTICS, *D*_p_, AND *D*_2_

Across all four‐taxon tests, we determined whether the number of tests supporting a higher incidence of introgression in the predicted direction—determined by geographical proximity or shared mating system—was greater than expected by chance, using sign tests performed in RStudio Team (2015). To assess if introgression is more prevalent between recently diverged lineages, we evaluated the association between *D*_p_ and genome‐wide genetic distance, using regression across all four‐taxon tests.

Our data also allowed us to evaluate several ancillary tests of these factors:

For geographical proximity, we could also determine if postspeciation introgression is associated with quantitative (rather than just qualitative) differences in proximity between heterospecific populations. To do so, for each trio we also determined the geographic distance (in km) between P1 and its nearest P3 accession, and between P2 and its nearest P3 accession (using our georeferenced location data; Text in the Supporting Information) to generate an estimate of their relative proximity to any population from P3 (i.e., the difference between these two distances; Table S4). For all four‐taxon geographic tests, we regressed *D*_p_ on this relative geographic distance estimate.

For mating system effects, one additional pattern we could test was whether mating system differences significantly affected the primary direction of introgression, as inferred from *D*_2_. To do so, we compared the number of cases where (SI → SC) versus (SC → SI), among all cases where differential introgression was inferred between lineages that had different mating systems (SI vs. SC), using a sign test.

Finally, we also evaluated evidence for the influence of potentially confounding factors on our analyses. In particular, to test whether our ability to detect introgression was affected by the magnitude of P1–P2 divergence (with reduced power to do so when P1 and P2 are very closely related), we assessed the relationship between *D*_p_ and this conspecific genetic distance (P1–P2) across all 17 trios. Because substantial gene flow between P1/P2 and P3 could also reduce the mean genetic distance calculated between these species, and thereby exaggerate the predicted negative relationship between genome‐wide genetic distance and *D*_p_, we also assessed the degree to which estimated introgression in our dataset could have influenced our estimates of genetic distance.

## Results

**Figure 3:**
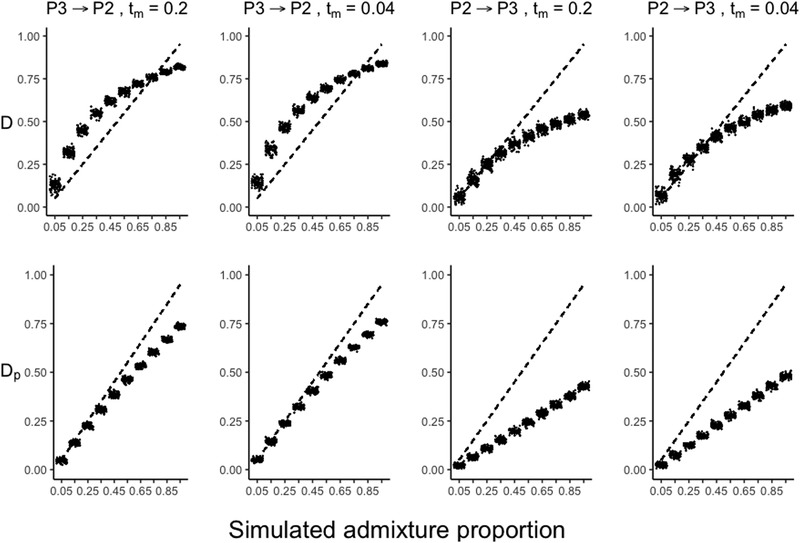
Simulated relationships of the *D* statistic (top row) and the *D*_p_ statistic (bottom row) with the admixture proportion. In each panel, the dashed line represents the expected relationship for a “perfect” estimator of the admixture proportion, and each dot represents the mean value of the statistic estimated from 100 replicate simulations. Each column represents a combination of simulated direction (P3 → P2 or P2 → P3) and timing (0.2N or 0.04N generations) of introgression.

We found that our proposed index of admixture proportion (*D*_p_) performed well as an estimator of the proportion of the genome originating from introgression. In simulations that varied the relative timing and the direction of introgression, inferred values of *D*_p_ tracked true values of admixture proportion closely (Fig. 3), although these values consistently fractionally underestimated known admixture. This underestimation was largest when the direction of introgression was P2 → P3 rather than P3 → P2—as expected because P2 → P3 introgression results in genealogies with shorter internal branches (Hibbins and Hahn 2019), allowing less time for ABBA substitutions to accumulate. The timing of introgression has comparatively little effect in all cases (Fig. 3). Nonetheless, simulated data fit expectations more closely when the admixture proportion was a smaller fraction of the genome; at 10% or less of the genome, the degree of underestimation of *D*_p_ is in the range of <1% for P3 → P2 introgression, and 2–3% for P2 → P3 introgression. At *D*_p_ of 0.05, which is greater than estimated for any trio in this study (see Results below), the magnitude of underestimation is <2% (Fig. 3). Moreover, under all examined conditions, *D*_p_ estimates varied linearly with the true value of introgression, indicating that the rank order of estimated admixture from *D*_p_ consistently agrees with the rank order of true admixture proportions. In comparison, the *D*‐statistic tends to overestimate the fraction of introgression in the P3 → P2 direction (as previously observed in Martin et al. 2015), and tracks the true value closely in the P2 → P3 direction (more closely than *D*_p_ at small values of introgression) until the proportion is approximately 50%. In addition, in all cases the value of *D* does not vary linearly with the admixture proportion (Fig. 3), as previously observed (Martin et al. 2015).

Using whole genome data from *Solanum*, we generated genome‐wide *D* estimates for 17 four‐taxon combinations, 14 that could address the effects of geographical proximity on introgression, and five addressing the effects of mating system variation (two of which were shared with the geographical set). All 17 combinations were used to assess the effect of genetic distance. Across all 17 tests, the genome‐wide average estimates of *D* ranged from –0.12 to +0.21, with 13 of these (76%) showing *D* values >0 (Table 1). Similarly, in each trio, the number of 100‐kb windows with positive *D* values exceeded those with negative *D* values in almost all trios, with the deficit or excess of +*D* windows in the same direction as the inferred genome‐wide average *D* (Table S3). The proportion of the genome estimated to be differentially introgressed (*D*_p_) ranged from 0.06% to 2.44% across trios (Table 2). Of the 15 four‐taxon tests with significant *D*, *D*_2_ tests inferred that 13 involved introgression from either P2 or P1 into P3, and two involved the alternative primary direction (Table 2).

**Table 1:** Introgression statistics for each analyzed trio (four‐taxon test). Trios in bold have *D*‐statistics that are significantly different than zero after Bonferroni correction. The order in which each species accession is listed corresponds to (P1, P2, P3). In all instances, we use the potato genome (*S. tuberosum*) as the outgroup. * denotes the two geographic trios that were also included in the mating system analyses. ^†^The P1 and P2 positions shown for this geographic trio are reversed in the mating system test. SD, standard deviation; S.E., standard error of mean; 95 lwr and 95 upr, lower and upper 95% confidence intervals, respectively. For geographic trios with nonoverlapping accessions, see Table S5

Species in trio	Accessions in trio	Mean *D*	*D* SD	*D* S.E.	95 lwr	95 upr	*P*‐value
Geographic trios							
**gal.gal.che**	**LA1044.LA0483.LA0746**	**−0.115**	**0.02**	0.000621111	**−0.154**	**−0.077**	<0.001
**arc.arc.pim**	**LA2172.LA2157.LA2147**	**0.035**	**0.012**	0.000369606	**0.012**	**0.058**	0.001
**pim.pim.neo**	**LA1375.LA1246.LA2133**	**−0.119**	**0.009**	0.000270316	**−0.136**	**−0.102**	<0.001
pim.pim.chi	LA1582.LA1933.LA1969	0	0.009	0.000289765	−0.018	0.018	0.493
**pim.pim.cor1**	**LA0400.LA1269.LA0118**	**0.08**	**0.009**	0.000277774	**0.063**	**0.097**	<0.001
**pim.pim.cor2**	**LA1617.LA1521.LA0118**	**0.176**	**0.008**	0.000266928	**0.159**	**0.192**	<0.001
**pim.pim.per1**	**LA1595.LA1341.LA1278**	**0.02**	**0.007**	0.000225502	**0.006**	**0.034**	0.002
**pim.pim.per2**	**LA1617.LA1269.LA1278**	**0.144**	**0.008**	0.000254186	**0.128**	**0.159**	<0.001
**pim.pim.hab**	**LA0417.LA0442.LA1777**	**0.083**	**0.008**	0.000255345	**0.067**	**0.099**	<0.001
**pim.pim.pen**	**LA1245.LA1269.LA1272**	**0.211**	**0.009**	0.000276221	**0.194**	**0.228**	<0.001
arc.arc.hab*†	**LA2172.LA2157.LA1718**	**−0.025**	**0.006**	0.000187304	**−0.037**	**−0.013**	<0.001
**hab.hab.neo**	**LA1777.LA1718.LA2133**	**0.049**	**0.008**	0.000259717	**0.033**	**0.065**	<0.001
hab.hab.cor*	**LA0407.LA1777.LA0118**	**0.018**	**0.006**	0.000203084	**0.005**	**0.03**	0.001
**hua.hua.hab**	**LA1983.LA1365.LA1718**	**0.06**	**0.006**	0.00019779	**0.048**	**0.073**	<0.001
Mating system trios							
**arcSI.arcSC.pimSC**	**LA2172.LA2157.LA0373**	**0.036**	**0.012**	0.000369758	**0.013**	**0.059**	0.001
habSI.habSC.pimSC	LA1777.LA0407.LA0373	0.009	0.012	0.000367901	−0.014	0.032	0.22
**perSI.perSC.pimSC**	**LA1278.PI128650.LA0373**	**0.042**	**0.008**	0.000249916	**0.026**	**0.057**	<0.001

**Table 2:** The proportion of the genome estimated to have differentially experienced introgression in each trio, calculated as (ABBA – BABA)/(ABBA + BABA + BBAA). Trios are listed in the same order as Table 1. Est. Prop. Genome, estimated proportion of the genome. * denotes the two geographic trios that were also included in the mating system analyses. ^†^The P1 and P2 positions shown for this geographic trio are reversed in the mating system test

Species in trio	Accessions in trio	Sum BBAA	Sum ABBA	Sum BBAA	Est. Prop. Genome	Direction of introgression	Donor mating system	Recipient mating system
Geographic trios								
gal.gal.che	LA1044.LA0483.LA0746	46,498	5681	7128	0.0243	P3 → P1	SC	SC
arc.arc.pim	LA2172.LA2157.LA2147	676,317	179,196	167,118	0.0118	P2 → P3	SC	SC
pim.pim.neo	LA1375.LA1246.LA2133	1,042,520	30,256	38,345	0.0072	P1 → P3	SC	SC
pim.pim.chi	LA1582.LA1933.LA1969	580,372	11,157	11,156	1.00 × 10^–6^	–	–	–
pim.pim.cor.1	LA0400.LA1269.LA0118	830,576	15,876	13,496	0.0027	P2 → P3	SC	SI
pim.pim.cor.2	LA1617.LA1521.LA0118	758,360	28,604	20,071	0.0105	P2 → P3	SC	SI
pim.pim.per.1	LA1595.LA1341.LA1278	1,378,868	20,202	19,415	0.0005	P3 → P2	SI	SC
pim.pim.per.2	LA1617.LA1269.LA1278	799,340	24,547	18,389	0.0073	P2 → P3	SC	SI
pim.pim.hab	LA0417.LA0442.LA1777	1,420,138	26,068	22,049	0.0027	P2 → P3	SC	SI
pim.pim.pen	LA1245.LA1269.LA1272	562,806	16,894	11,016	0.0099	P2 → P3	SC	SI
arc.arc.hab*†	LA2172.LA2157.LA1718	961,051	113,676	119,508	0.0048	P1 → P3	SI	SI
hab.hab.neo	LA1777.LA1718.LA2133	1,209,794	73,866	67,047	0.005	P2 → P3	SI	SC
hab.hab.cor*	LA0407.LA1777.LA0118	1,085,238	80,588	77,829	0.0022	P2 → P3	SI	SI
hua.hua.hab	LA1983.LA1365.LA1718	491,701	120,471	106,775	0.019	P2 → P3	SI	SI
Mating system trios								
arcSI.arcSC.pim.SC	LA2172.LA2157.LA0373	670,146	180,425	167,904	0.0122	P2 → P3	SC	SC
habSI.habSC.pimSC	LA1777.LA0407.LA0373	1,327,687	90,995	89,367	0.001	–	–	–
perSI.perSC.pimSC	LA1278.PI128650.LA0373	566,678	122,803	112,923	0.0123	P2 → P3	SC	SC

**Figure 4:**
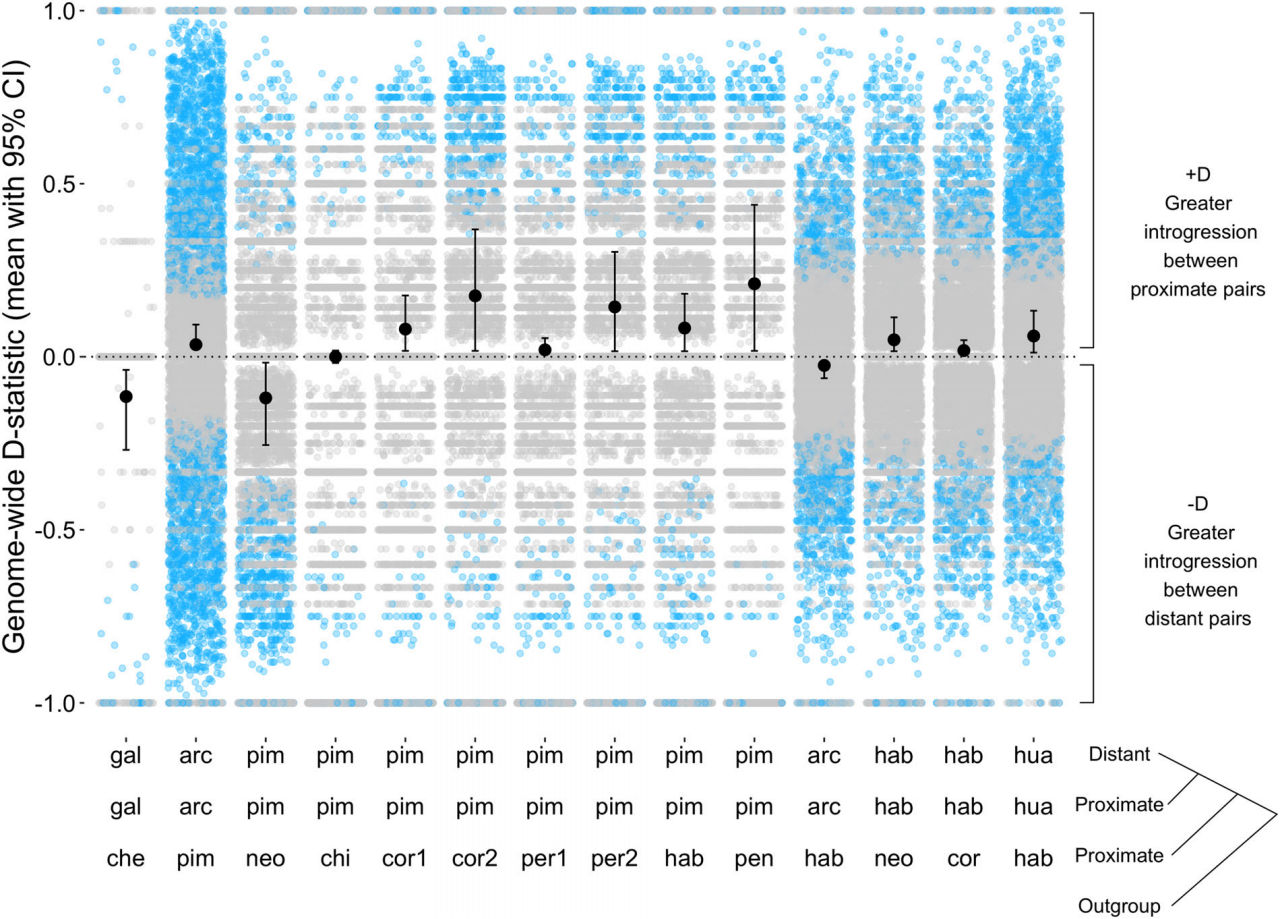
Effect of geographical proximity on introgression. For each geographic trio, the plot shows genome‐wide mean *D* values and 95% confidence intervals, as well as *D*‐statistic estimates from individual 100‐kb windows with >20 SNPs (gray circles: individual window *D* values that were outliers [blue] or not outliers [gray]). The order of trios (from L to R) along the *X*‐axis is from least divergent to most divergent (least to greatest mean genetic distance between P1/P2 and P3), and corresponds to the order in which they are listed (top to bottom) in Table 1, where accession IDs are specified. The two geographic trios that are also used in the mating system analyses are arc.arc.hab (LA2172.LA2157.LA1718) and hab.hab.cor (LA0407.LA1777.LA0118). For all trios, our outgroup is potato (*S. tuberosum*).

Using these data, our tests for systematic effects of geography, mating system, and genetic distance suggest that each factor affects the prevalence of introgression, but that our power to detect these is limited by the number of comparisons currently available. First, for our tests of geographical proximity, of 14 testable four‐taxon combinations, 10 had average *D* values significantly greater than zero—indicating that our geographically closer lineages (P2 and P3) share a higher proportion of sites—whereas three were significantly less than zero (Figure 4; Table 1; one value did not differ from zero). Although this trends in the predicted direction, a two‐sided sign test indicated that the number of significantly positive (10/13, or 77%) versus negative mean values of *D* was not different (*P* = 0.092). Because there is nonindependence in our dataset (i.e., some individual accessions/genome sequences are used in more than one four‐taxon test), we also evaluated the influence of this nonindependence by paring our dataset down to trios (of P1, P2, P3) that only used unique accessions. Because our goal is to test very recent histories of differential introgression, and these trios only use each P1/P2 contrast once, they only sample any recent history of differential introgression involving these specific accessions once. Our 13 trios with significant *D*‐statistics can be reduced to 13 alternative combinations of seven trios that share no accessions in common (Table S5). In each alternative combination, trios with positive *D* exceeded those with negative *D* (i.e., introgression is consistently more frequently when populations of a species pair were geographically closer versus more distant); nonetheless this directionality is nonsignificant in each of the reduced datasets (Table S5a) as the two‐sided sign test is underpowered to detect a difference in direction when *n* = 7. Finally, across all 14 four‐taxon tests, *D*_p_ was not significantly associated with the relative geographical proximity of the P1 versus P2 population to the closest population from the P3 species (*R*‐squared = 0.079, *P*‐value = 0.328, Fig. S1), suggesting no evidence for a quantitative relationship between relative geographical proximity and the amount of inferred introgression.

**Figure 5:**
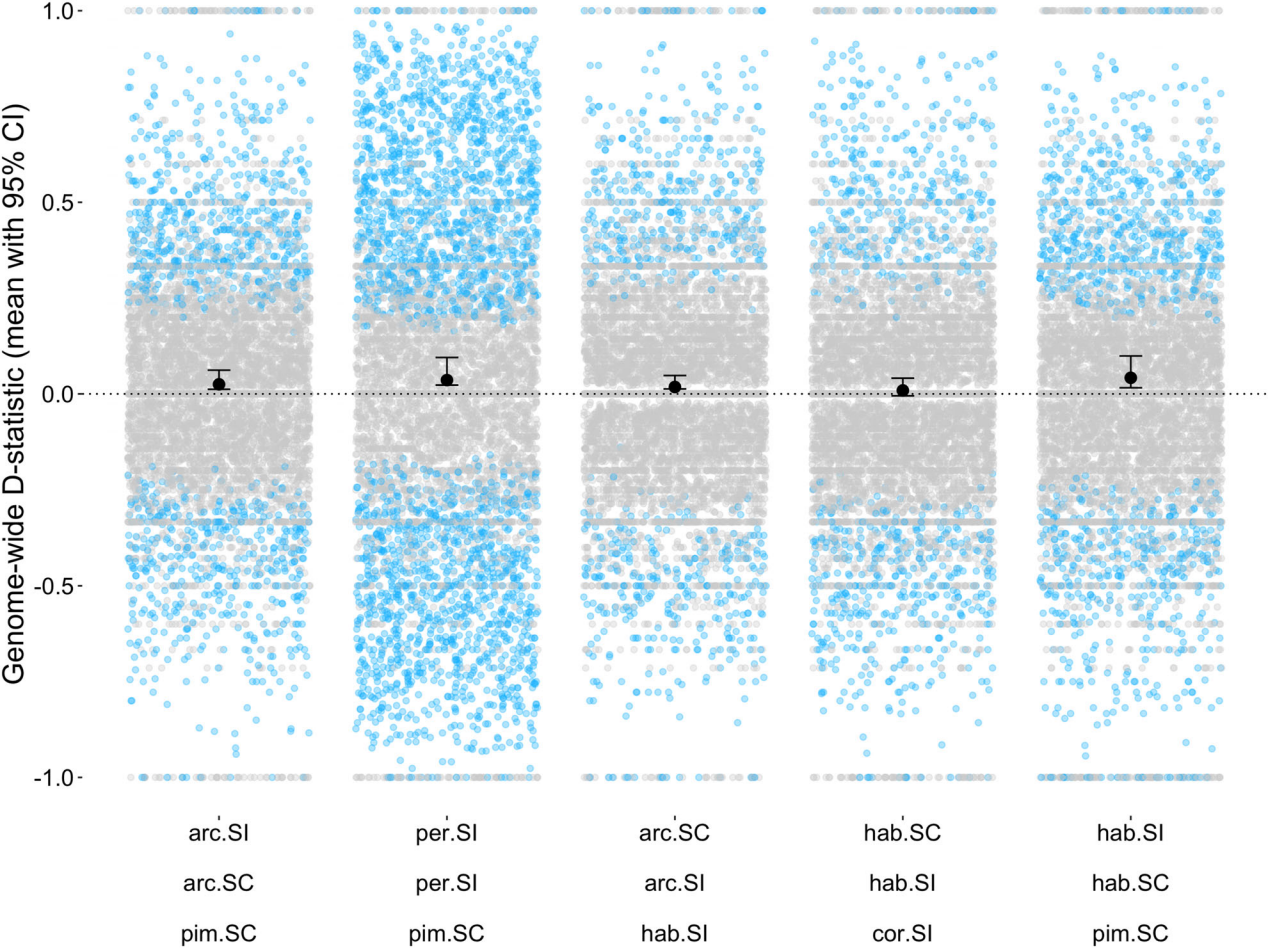
Effect of mating system differences on the observed direction of introgression. For each mating system trio, the plot shows genome‐wide mean *D* and 95% confidence intervals, as well as *D*‐statistic estimates from individual 100‐kb windows with >20 SNPs (individual window *D* values that were outliers [blue] or not outliers [gray]). The order of trios (from L to R) along the *X*‐axis is from least divergent to most divergent (least to greatest mean genetic distance between P1/P2 and P3). Accession IDs are specified are specified in Table 1. The two mating system trios that are also used in the geographic analyses are arcSC.arcSC.habSI (LA2172.LA2157.LA1718) and habSC.habSC.corSI (LA0407.LA1777.LA0118). For all trios, our outgroup is potato (*S. tuberosum*).

Second, for our evaluation of mating system effects, of five testable trios (three new trios and two trios that were also used in the geographic tests; see Table 1), four genome‐wide mean *D* values were significant in the expected direction, whereas one was not significantly different from zero (Table 1; Fig. 5). A two‐sided sign test comparing significantly positive (4/4, or 100%) versus negative (0) cases returned the smallest *P*‐value that can be obtained when *N* = 4 (*P* = 0.125), suggesting some evidence that introgression might be less constrained between lineages that share the same versus different mating system. Note that two of these cases are shared with the geographical set (as indicated in Table 1), but one of these requires switching the orientation of accessions in the P1 and P2 positions to test this alternative factor. Indeed, although this specific trio (arcLA2172.arcLA2157.habLA1718; Table 1) supports our a priori mating system hypothesis, it acts against our hypothesis based on relative geographic proximity, reiterating that geographic and mating system factors are not consistently associated within our dataset (Methods). Results were also not affected by which specific accession was used in the P3 position for the mating system trios (see Text in the Supporting Information).

In terms of a relationship more broadly between the primary direction of introgression and mating system variation, in the 15 trios where we evaluated *D*_2_ (Table 2), seven of these involved differential exchange between lineages with a different mating system (SI vs. SC). Of these, five cases inferred introgression from the SC lineage into the SI lineage, whereas two inferred the other direction. This difference is not significant (*P* = 0.453) but suggests some evidence that SC lineages are more likely to act as the donor lineage in these cases.

**Figure 6:**
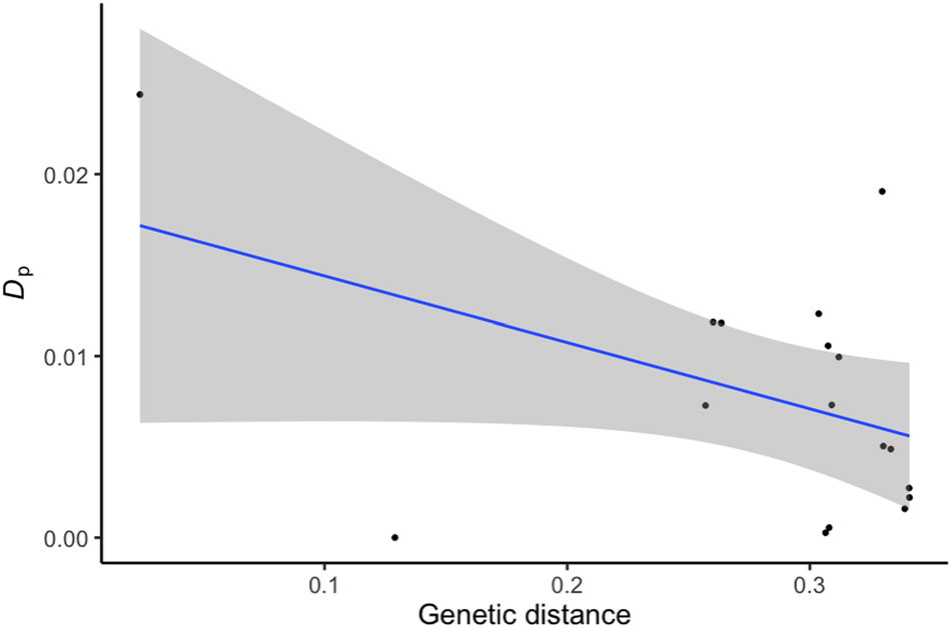
The relationship between genome‐wide *D*_p_ and the average genetic distance (% divergence across all sites) between P1/P2 and P3 species for 17 trios (*R*‐squared = 0.20, *P*‐value = 0.071).

Finally, we detected a marginal negative relationship between the estimated amount of differential introgression (*D*_p_) and increasing evolutionary divergence between P1/P2 and P3 species (*R*‐squared = 0.20, *P*‐value = 0.071, Fig. 6 and Table S6), suggesting some evidence that the propensity for introgression is higher among species pairs that are less evolutionarily divergent. In contrast, the absolute value of *D* was unrelated to the mean genetic distance between the focal species in each trio (*R*‐squared = 0.001, *P*‐value = 0.882, Fig. S2). *D*_p_ was unrelated to P1–P2 genetic distance across all trios (*r* ^2^ = 0.025, *P* = 0.539), indicating that our ability to detect introgression was not strongly influenced by how recently the P1/P2 conspecific accessions had a shared common ancestor. Finally, we note that the levels of introgressive hybridization we estimate here (<2.5% of the genome; Table 2) are not sufficient to substantively influence our estimates of genome‐wide genetic distance between P1/P2 and P3, and thereby influence (amplify) a negative relationship between *D*_p_ and genetic distance. For instance, for estimates of genetic distance typical of the trios here (4% or less; Table S6), these admixture proportions will only reduce genome‐wide genetic distance estimates by <0.1% divergence.

## Discussion

The prevalence of introgression is one pattern emerging from contemporary genome‐wide studies in many groups of closely related species, including in groups not traditionally associated with postspeciation gene flow (Mallet et al. 2016; Jones et al. 2018; Taylor and Larson 2019). However, there have been few attempts to systematically assess the influence of different factors in shaping the frequency and extent of this gene flow. Here, we used directionally structured, four‐taxon ABBA–BABA tests and statistics derived from these to examine the influence of three factors—genetic distance, geographical proximity, and mating system differences—on genome‐wide patterns of introgression among wild tomato species. We found that recent introgression was commonly detected among these species, but that the estimated fraction of the genome differentially introgressed between species was modest, and the prevalence of postspeciation introgression varied—albeit weakly—with aspects of all three biological factors evaluated here. These findings have interesting implications for interpreting the contexts in which introgression might play a role in shaping evolutionary trajectories in this and other similar clades, and for assessing the potential contribution of introgression to adaptive phenotypic evolution.

### RECENT INTROGRESSION OCCURS FREQUENTLY BUT IS MODEST IN SCOPE AMONG WILD TOMATOES

Our analysis indicates that, among wild tomato lineages, postspeciation gene exchange is prevalent: of 17 total four‐taxon tests across all our analyses, 15 had mean *D* values significantly different from zero. Prior studies have detected evidence for introgression among specific wild tomato lineages (Pease et al. 2016; Beddows et al. 2017), and our findings expand and illuminate these observations in several key respects. First, our analyses preferentially assessed evidence for recent, rather than more ancient (Pease et al. 2016) introgression events because in every case we contrasted populations (P1 and P2) from a single species when looking for evidence of introgression with a second species (P3). Accordingly, any inferred introgression must have occurred after the evolutionary split of these two (P1 and P2) conspecific populations. Despite this, we find repeated evidence that populations from different species have exchanged genes recently, including species that are estimated to have diverged >2 million years ago (e.g., *S. pimpinellifolium* and *S. pennellii*; Pease et al. 2016).

Our results suggest there is broad potential for cross‐species hybridization across the clade, a finding consistent with other observations that indicate premating isolation is likely to be incomplete among lineages in nature: all species share general floral morphology (rotate, yellow, five‐petaled flowers), all are buzz‐pollinated, and multiple species pairs are found in sympatry (Rick 1950). Nonetheless this finding is intriguing as few natural hybrids have been observed in the wild in this group (Taylor 1986), and some of these species are known via crossing and genetic studies to express moderate to strong postmating and postzygotic reproductive isolation under lab conditions (especially species in different subclades, including *S. pennellii* and *S. habrochaites* with species from the red‐fruited clade including *S. pimpinellifolium*; Moyle and Nakazato 2008, 2010; Hamlin et al. 2017). These later‐acting barriers might be important in limiting the amount of introgression that results from hybridization events.

Indeed, a second general observation of our analysis is that despite evidence for relatively frequent hybridization, the amount of the genome exchanged between species is likely to be limited: the proportion of the genome estimated to be differentially exchanged between species is on the order of 0.06% to approximately 2.5% (Table 2). In addition to being limited in scope, our data also suggest that introgression varies across the genome. For example, when window‐based *D*‐statistics (Fig. S3) or alternative site topologies (Figs. 4 and 5) are examined chromosome‐by‐chromosome within each four‐taxon test, in most cases introgression is inferred on some chromosomes but not others; this variation among chromosomes might be due to variation in the presence of loci contributing to reproductive isolation. Similarly, exploring *D* values within individual 100‐Kb windows (Figs. 3 and 4; Table S3) suggests that there are numerous locations in the genome consistent with an outlier *D* value, rather than genome‐wide average *D* being explained by a few large introgression blocks that are concentrated in specific genomic regions. Overall, the amount and distribution of inferred introgression suggest that current species reproductive barriers are sufficiently incomplete to allow detectable recent introgression among diverged species in the field, but also that genomes are not completely or uniformly porous to gene flow among lineages, even in cases where there is an opportunity for gene exchange.

### INTROGRESSION IS RELATED TO SPATIAL PROXIMITY, MATING SYSTEM DIFFERENCES, AND OVERALL GENETIC RELATEDNESS BETWEEN SPECIES PAIRS

Importantly, our analyses also allowed us to explicitly assess the influence of several factors on these detected patterns of introgression. We found that repeated patterns of recent postspeciation hybridization were weakly associated with all three factors, although our power to discriminate these was limited by the whole‐genome data currently available for this analysis. In terms of overall genetic relatedness among species, we observed a marginal negative association between the magnitude of evolutionary divergence (genetic distance), and the amount of inferred introgression, across all trios. Species are expected to accumulate reproductive isolation with increasing evolutionary divergence (Coyne and Orr 1997), and this pattern has been observed among wild tomatoes for loci involved in postzygotic reproductive isolation (hybrid pollen and seed sterility; Moyle and Nakazato 2010), suggesting that introgression should become attenuated with increasing evolutionary age among species. Here, because our analysis was limited to accessions for which we had whole‐genome data and to trios that meet our a priori criteria, this relationship largely examined (P1/P2, P3) species pairs with intermediate levels of divergence (∼3–4%), and only a single (P1/P2, P3) species pair where genetic distance was <1% (Table S6). The lack of more closely related species might have affected our power to detect a strong quantitative relationship here. Apart from low power, several other factors could also act to weaken this expected relationship. For instance, the total number of loci estimated to contribute to postzygotic isolation in this group is relatively modest, even among the oldest species pairs (Moyle and Nakazato 2008, 2010), and mean sequence divergence between all lineages analyzed here is low—0.02‐0.3%—consistent with the recent, rapid origin of species in this clade (Pease et al. 2016). Both could reduce the strength of a monotonic relationship between admixture proportion and evolutionary distance, because they indicate that modest gene flow might persist among even the most distant pairs of species in this clade. Using data from a very broad range of taxa, a recent meta‐analysis inferred that divergence of just a few percent results in barriers that can effectively suppress gene flow (Roux et al. 2016) indicating that genetic divergence can strongly determine introgression, at least beyond some threshold at which isolating barriers are sufficiently strong. The data presented here suggest that wild tomato species have not yet exceeded this threshold, and that genetic relatedness is likely just one factor that contributes to shaping recent introgression in this group.

Geographical proximity is another such factor. Our data suggest that relative geographic proximity between populations of different species often, but not always, increases their propensity for introgression. This is consistent with the expectation that the simple opportunity for reproductive contact influences, but does not determine, that postspeciation gene exchange will be more prevalent. Similarly, although we currently have too few four‐taxon tests to draw definitive conclusions, we found some indication that mating system differences could be associated with two general effects. First, our data suggest that the propensity of introgression is more likely between lineages that share a mating system, at least as far as this is captured by differences between SI and SC. Second, in cases where introgression is inferred between lineages with different mating system, most often the direction of introgression is from SC into SI lineages. The first observation is consistent with our a priori prediction. The second suggests evidence in support of the theoretical expectation that SC lineages might be particularly resistant to alleles from more outbreeding lineages. In this direction of introgression, the genetic load that persists in outbreeders can be exposed (via homozygosity) in a more inbreeding context, so that donor alleles are expected to have stronger deleterious fitness effects in general. Moreover, smaller *N_e_* in the recipient population reduces the effectiveness of recombination at disassociating these from other nondeleterious loci before they are purged (Ruhsam et al. 2011; Brandvain et al. 2014). Although this reduces the chance of SI alleles persisting in a recipient SC population, neither of these effects are strong in the reciprocal SC → SI direction of gene flow. This specific direction of introgression is also supported by observations from detailed case studies in other plant groups (e.g., *Mimulus*; Brandvain et al. 2014). Within the limitations of the data available here, then, mating system differences do appear to potentially influence both the propensity for recent introgression, and the direction it might take. Nonetheless, because there are clear predictions about associations between mating system, genetic load, the efficacy of selection, and the propensity and direction of introgression (Lande and Schemske 1985; Charlesworth et al. 1990; Busch 2005; Harris and Nielsen 2016; Juric et al. 2016), testing the generality of these effects with a larger set of comparisons remains a goal in the future.

Finally, as our preliminary inferences suggest, the conditions that generally favor or prevent gene flow between species will likely vary depending on the biological features of different systems. Here, we evaluated several factors that are likely influential in wild tomato populations, but did not assess other biological components—such as variation in the relative local density of species or the magnitude of overlap in species ranges—that might also influence gene flow in wild systems, but about which we have less a priori information. For example, some previous analyses have assessed factors influencing general introgression patterns with different approaches. For example, Winger (2017) evaluated the relationship between introgression and plumage differentiation for 16 lineages of Andean cloud forest birds within a geographically and ecologically structured study. He found evidence for introgression across a geographic barrier between lineage pairs with uniform plumage patterns, but not between pairs with divergent plumage. This suggests that different patterns of sexual selection might determine whether and when introgression is expected, although alternative explanations, including more time since divergence between plumage‐differentiated pairs, could not be excluded in this case. Clearly, additional tests of the ecological, reproductive, and historical factors most strongly predictive of postspeciation gene flow will be helpful in evaluating how these might or might not differ between major groups of organisms.

### EVALUATING INTROGRESSION AS AN IMPORTANT EVOLUTIONARY FORCE

Our analyses join a growing consensus of studies that suggest gene flow among distinct lineages might be common, especially those that have rapidly diverged, have incomplete isolating barriers, and that maintain some regions of geographical overlap (The et al. 2012; Brawand et al. 2014; Jónsson et al. 2014; Lamichhaney et al. 2015). Our findings also reaffirm that although genetic exchange in any particular instance is likely to be influenced by both ecological and genomic contexts, only via systematic tests of introgression patterns across multiple cases can we start to assess the major determinants of postspeciation gene flow. One of our goals here was to demonstrate that structured a priori tests and associated analyses that draw on ABBA–BABA statistics provide one framework for assessing the influence of general factors on the frequency and amount of postspeciation introgression, when applied across multiple species pairs. Moreover, most of the specific analyses proposed here require comparatively modest a priori information to implement, beyond what is already required to generate conventional *D*‐statistics. For example, *D_p_* requires only one sample per lineage, making it comparable to other coalescent‐based methods for quantifying the magnitude of introgression, such as F_4_ ancestry estimation (Patterson et al. 2012), PhyloNet (Than et al. 2008; Wen et al. 2018), and the *f*_d_ statistic (Martin et al. 2015). It has similar behavior to *f*_d_ estimated in genomic windows, but can be used to quantify the genome‐wide rate of introgression. Further, by requiring only biallelic site counts for a four‐taxon tree, *D*_p_ has more lenient data requirements than the F_4_ ratio (which requires a five‐taxon tree and known demographic history) or PhyloNet (which requires gene trees as input, or priors in the case of Bayesian inference). The increasing availability of whole‐genome data from multiple closely related species therefore makes the implementation of these kinds of approaches increasingly accessible in the near future.

With such tests, we demonstrated here that recent introgressive hybridization is common among wild tomatoes, but results in relatively small admixture proportions. Whether introgression contributes substantially to shaping the evolution of these lineages remains to be determined. Even very restricted gene flow can be consistent with “adaptive” introgression—the movement of alleles among species that increase fitness in their new recipient lineage (Suarez‐Gonzalez et al. 2018). Indeed, several of the best cases of apparently adaptive introgression involve small chromosomal regions (e.g., mimicry loci in *Heliconius*; high altitude adaptation in ancestral human populations; Huerta‐Sánchez et al. 2014; The Heliconius Genome Consortium et al. 2012). Although not a primary goal of our analyses here, we did not observe any obvious analogous cases at known mating‐system loci in our genomes (see Text in the Supporting Information; Fig. S4). For now, the observation that a low level of gene exchange frequently occurs between lineages suggests that introgression could be a significant source of adaptive genetic variation, certainly in comparison to lineages where there is no evidence of gene flow. Regardless, by going beyond individual cases to generate general and quantitative evaluations, similar analyses of this kind should clarify both the frequency, extent, and main determinants of postspeciation introgression across a diversity of organisms and contexts, and the potential contribution of introgression as an engine of evolution.

Associate Editor: Z. Gompert

## Supporting information


**Figure S1**. Relationship between the relative difference in geographic distance of P1 and P2 from a heterospecific P3 population and the proportion of introgression as calculated from *D_p_*.

**Figure S2**. Relationship between the average genome‐wide genetic distance, for each analyzed trio, and the absolute value of D.

**Figure S3**. Chromosome‐by‐chromosome (x‐axis) distribution of D‐statistic (y‐axis) estimates from individual 100kb windows (black circles: window D not significantly different than zero; red circles: window D significantly different than zero), for each geographic trio from windows with only > 20 SNPs. See Table 1 for abbreviations.

**Figure S4**. The distribution of inferred tree topologies across the genome, for each trio in our mating system tests.

**Figure S5**. The distribution of inferred tree topologies across the genome for each trio in our geographic tests.

**Table S1**. Introgression statistics for each analyzed trio (four‐taxon test).
**Table S2**. Estimated proportion of genome introgression (*D*_p_) and the direction of introgression (from D2 tests) for each analyzed trio (four‐taxon test).
**Table S1**. Species, accession, associated ID for the three different sequencing projects and the species used in this analysis.
**Table S2**. Pairwise geographic distances among P1, P2, and P3 accessions used in all analyzed trios (four‐taxon tests).
**Table S3**. Number of windows examined for each trio for both the entire dataset and the reduced dataset (i.e. only windows for which number of informative sites were greater than 20).
**Table S4**. Geographic distance between either P1 or P2 and their closet accession from the species used in the P3 position.
**Table S5a**. The unique combinations (independent accessions) from 13 choose 7 with the associated D statistic for each trio.
**Table S5b**) Each row is a permutated combination from 13 to choose 7 trios, where each column is one set of trios.
**Table S6**. Estimated genome‐wide genetic distance (expressed as sequence difference) for each geographic and mating system trio.
**Table S7a**. Empirical *D*_2_ values, summary of simulated null distributions, and p‐values for each trio.
**Table S7b**. Demographic parameters used to simulate a null distribution of D2 statistics for each trio.
**Table S7c**. Species‐level heterozygosity estimates from Pease et al. (2016).
**Table S8a**. Output of genes found in regions with 10 (or greater windows), which show alternative site pattern preference for ArcSI and Arc SC (chromosome1; range = 43800000..44900000).
**Table S8b**. Output of genes found in regions with 10 (or greater windows), which show alternative site pattern preference for ArcSI and Arc SC (chromosome1; range = 61200000..62700000).
**Table S8c**. Output of genes found in regions with 10 (or greater windows), which show alternative site pattern preference for ArcSI and Arc SC (chromosome3; range = 29300000..30900000).
**Table S8d**. Output of genes found in regions with 10 (or greater windows), which show alternative site pattern preference for ArcSI and Arc SC (chromosome3; range = 33500000..35700000).
**Table 8e**. Output of genes found in regions with 10 (or greater windows), which show alternative site pattern preference for ArcSI and Arc SC (chromosome12; range = 54000000..55400000).
**Table S8f**. Output of genes found in regions with 10 (or greater windows), which show alternative site pattern preference for ArcSI and Arc SC (chromosome12; range = 58300000..60800000).
**Table S8g**. Output of genes found in regions with 10 (or greater windows), which show alternative site pattern preference for Hab SI and Hab SC (chromosome1; range = 54900000..57100000).
**Table S8h**. Output of genes found in regions with 10 (or greater windows), which show alternative site pattern preference for Per SI and Per SC (chromosome12; range = 78200000..80200000).
**Table 8i**. Output of genes found in regions with 10 (or greater windows), which show alternative site pattern preference for Arc SC, Arc SI and Hab SI (chromosome 1; range = 59500000..60600000).
**Table 8j**. Output of genes found in regions with 10 (or greater windows), which show alternative site pattern perference for Hab SC, Hab SI and Cor SI (chromosome 1; range = 55600000..56700000).
