# Supplementary figures and images for: Assessing biological factors affecting postspeciation introgression
Source: Evol Lett. 2020 Feb 28;4(2):137–54. doi: 10.1002/evl3.159 (PMC7156103; doi:10.1002/evl3.159)

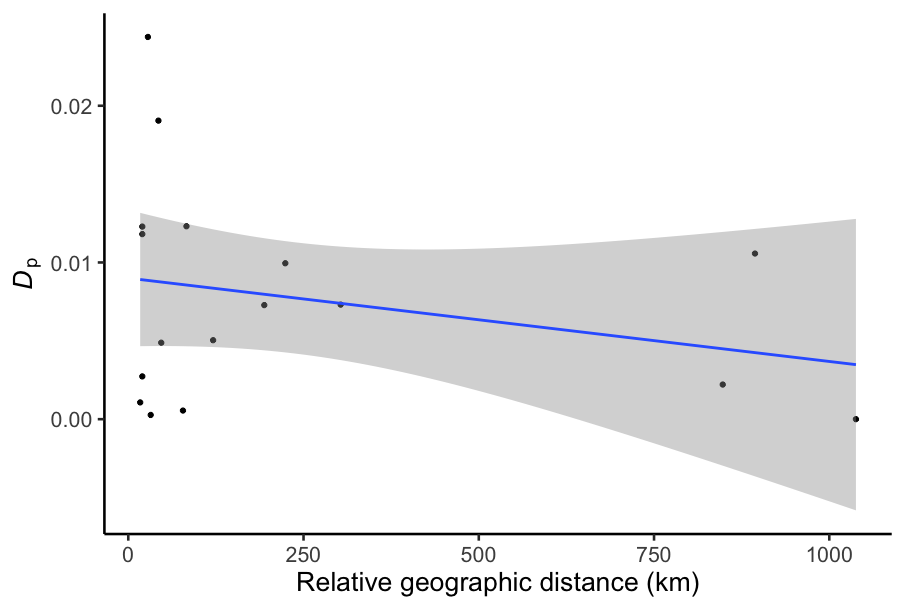

Supplement: Supplementary file 1 — Figure S1. Relationship between the relative difference in geographic distance of P1 and P2 from a heterospecific P3 population and the proportion of introgression as calculated from Dp. [file EVL3-4-137-s001.png]

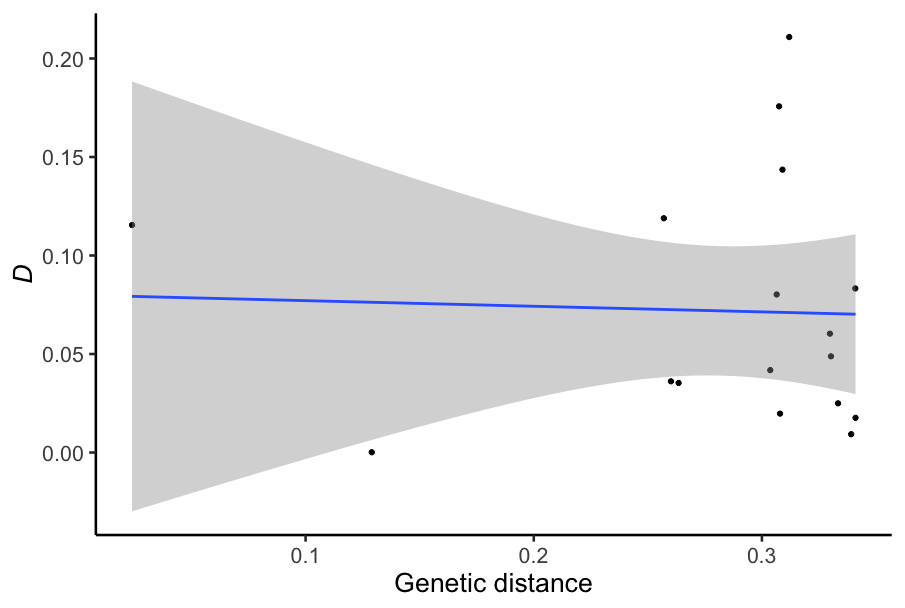

Supplement: Supplementary file 2 — Figure S2. Relationship between the average genome‐wide genetic distance, for each analyzed trio, and the absolute value of D. [file EVL3-4-137-s002.png]
