# Supplementary material for: Assessing biological factors affecting postspeciation introgression
Source: Evol Lett. 2020 Feb 28;4(2):137–54. doi: 10.1002/evl3.159 (PMC7156103; doi:10.1002/evl3.159)
Supplement: Supplementary file 3 — Figure S3. Chromosome‐by‐chromosome (x‐axis) distribution of D‐statistic (y‐axis) estimates from individual 100kb windows (black circles: window D not significantly different than zero; red circles: window D significantly different than zero), for each geographic trio from windows with only > 20 SNPs. See Table 1 for abbreviations. [file EVL3-4-137-s003.pdf]

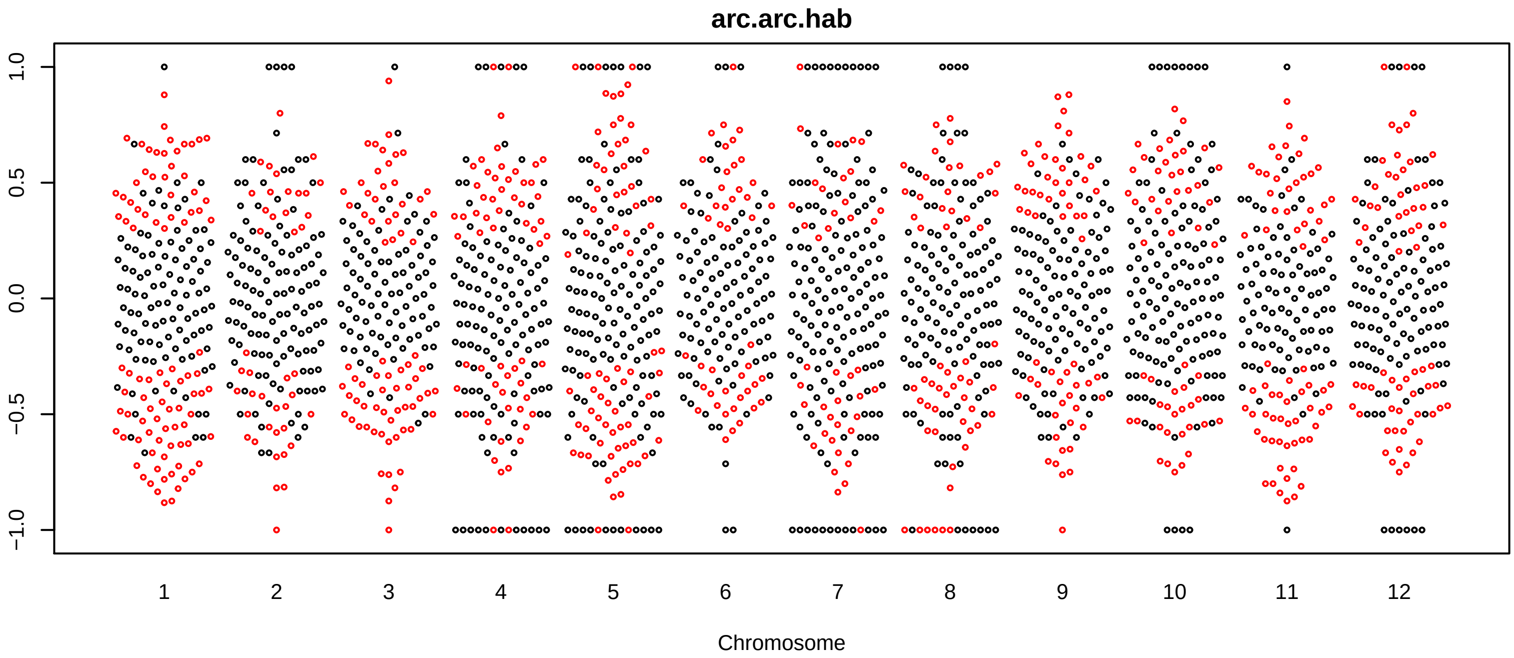
arc.arc.hab
1.0
0.5
0.0
−0.5
−1.0
1 2 3 4 5 6 7 8 9 10 11 12
Chromosome

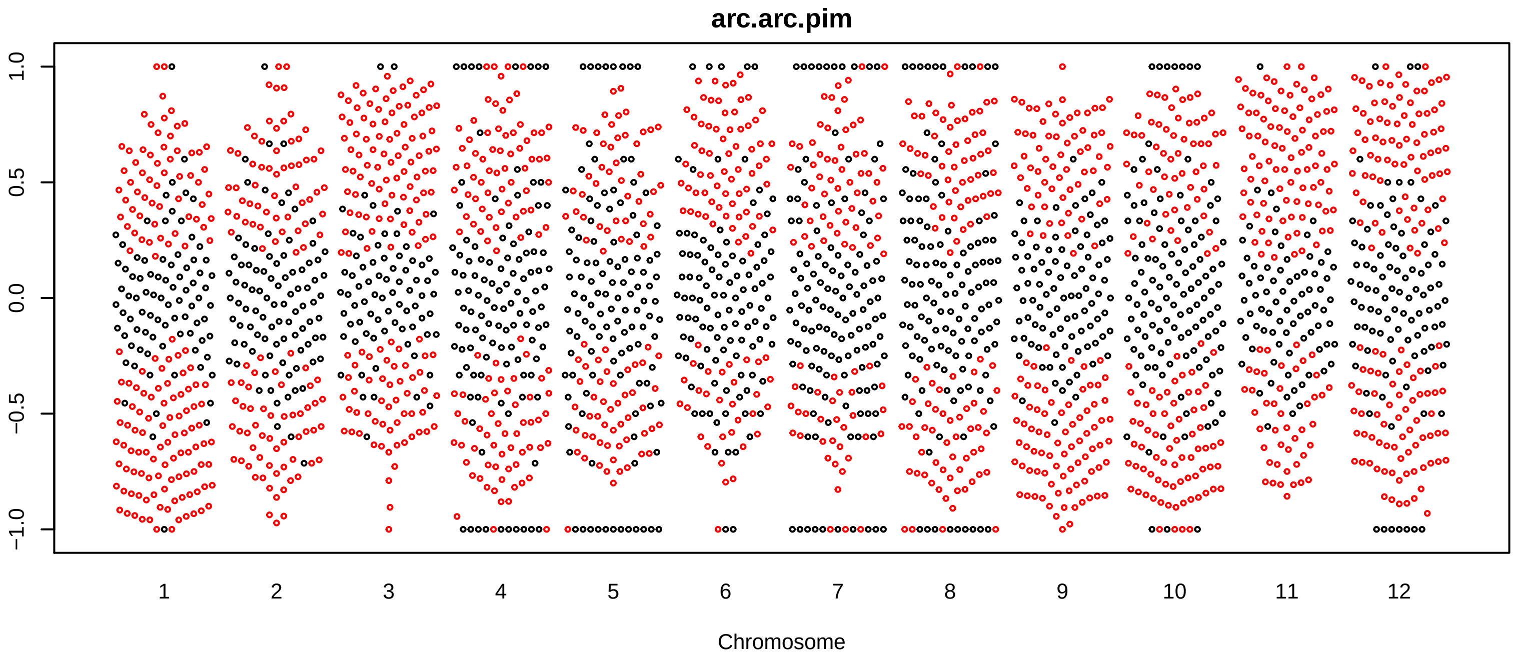
arc.arc.pim
1.0
0.5
0.0
−0.5
−1.0
1 2 3 4 5 6 7 8 9 10 11 12
Chromosome

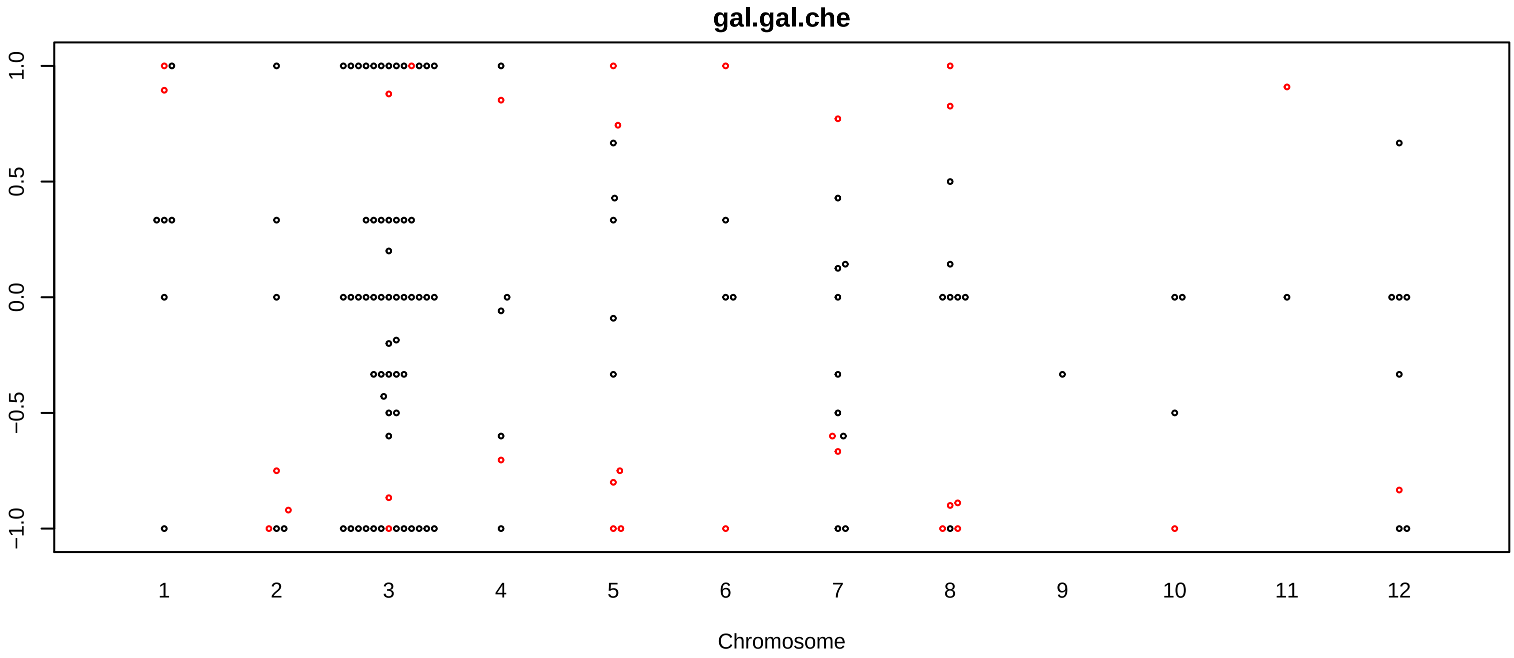
gal.gal.che
1.0
0.5
0.0
−0.5
−1.0
1 2 3 4 5 6 7 8 9 10 11 12
Chromosome

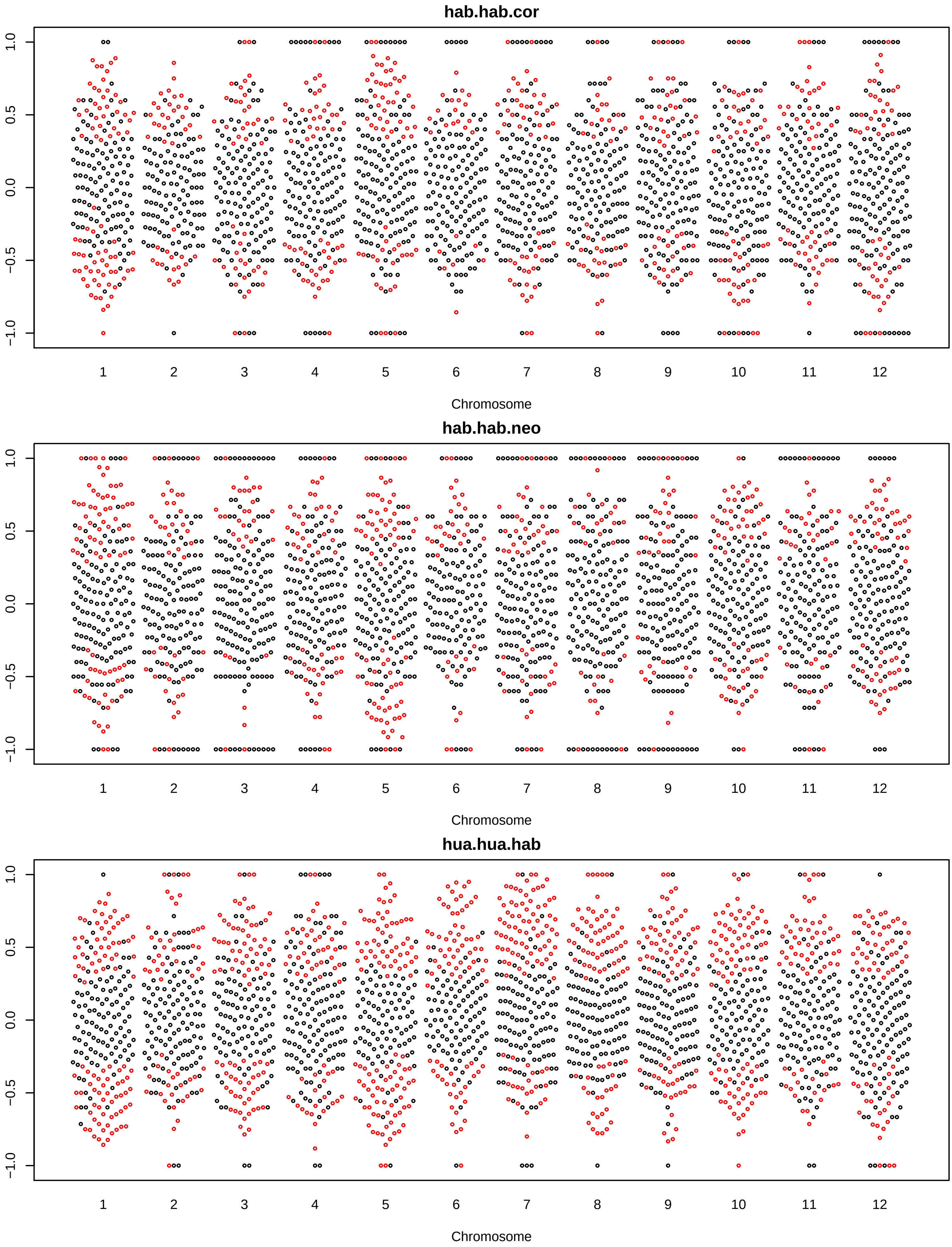

hab.hab.cor
hab.hab.neo
hua.hua.hab
Chromosome
1.0
0.5
0.0
−0.5
−1.0
1
2
3
4
5
6
7
8
9
10
11
12

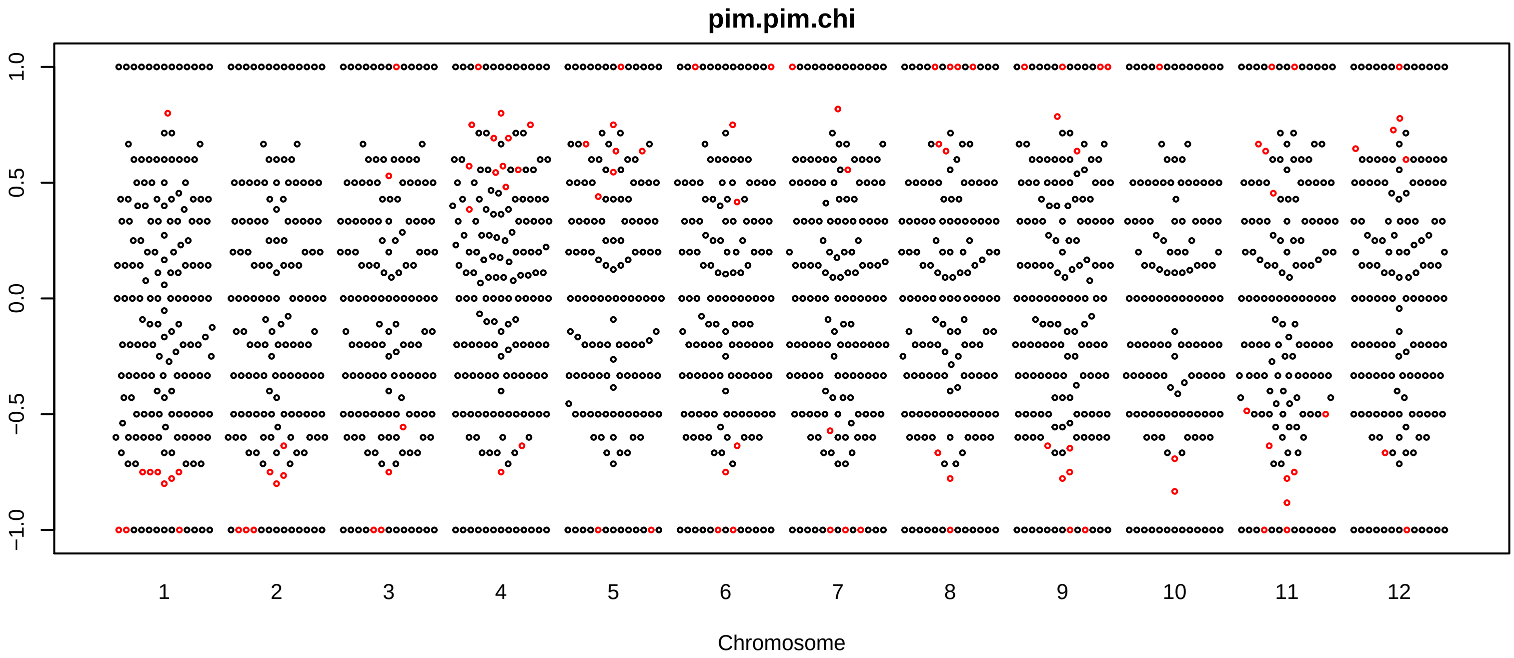
pim.pim.chi
1.0
0.5
0.0
−0.5
−1.0
1
2
3
4
5
6
7
8
9
10
11
12
Chromosome

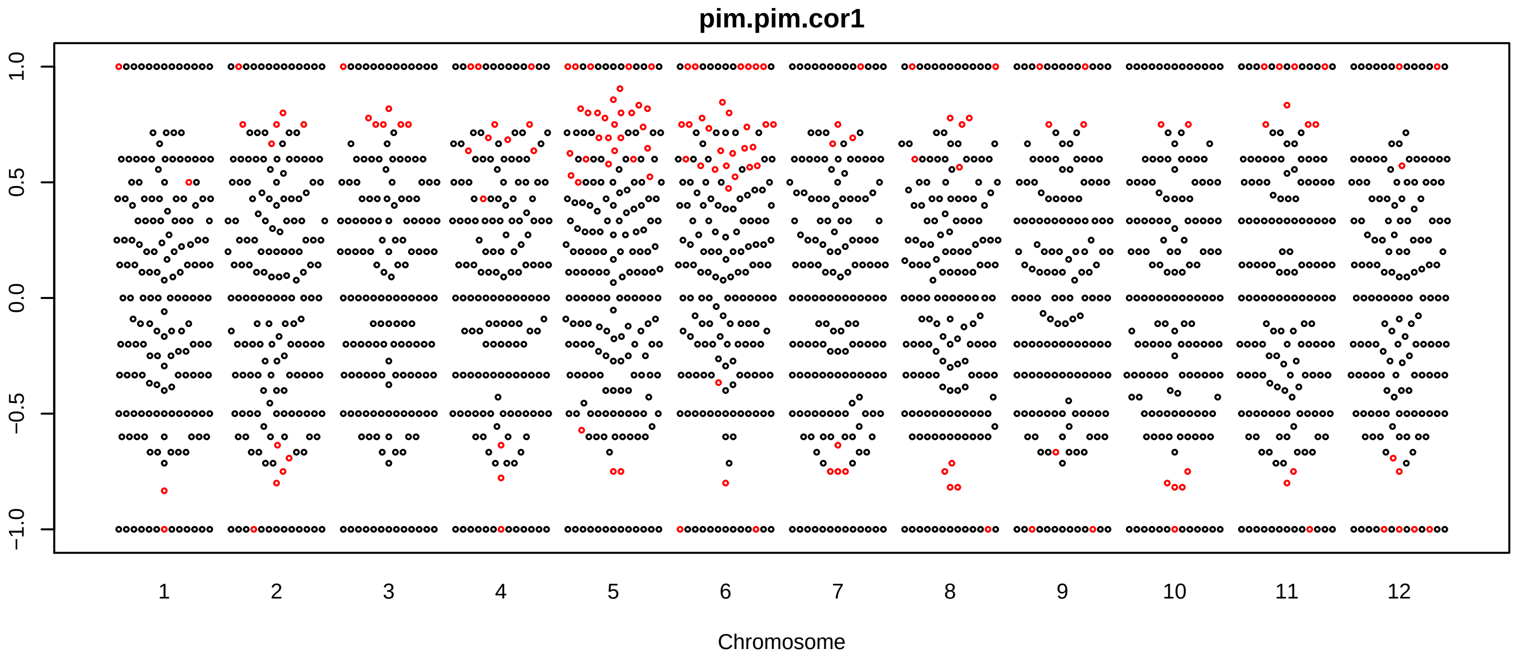
pim.pim.cor1
1.0
0.5
0.0
−0.5
−1.0
1
2
3
4
5
6
7
8
9
10
11
12
Chromosome

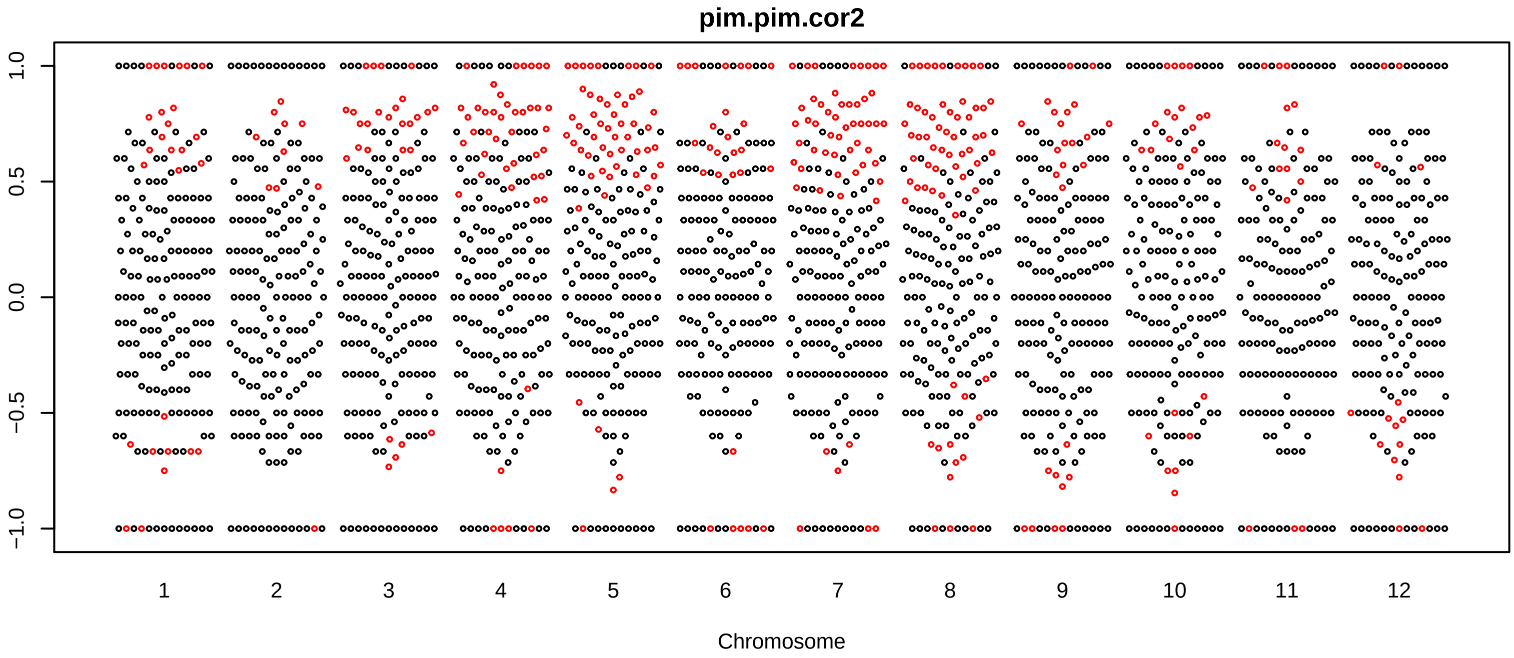
pim.pim.cor2
1.0
0.5
0.0
−0.5
−1.0
1
2
3
4
5
6
7
8
9
10
11
12
Chromosome

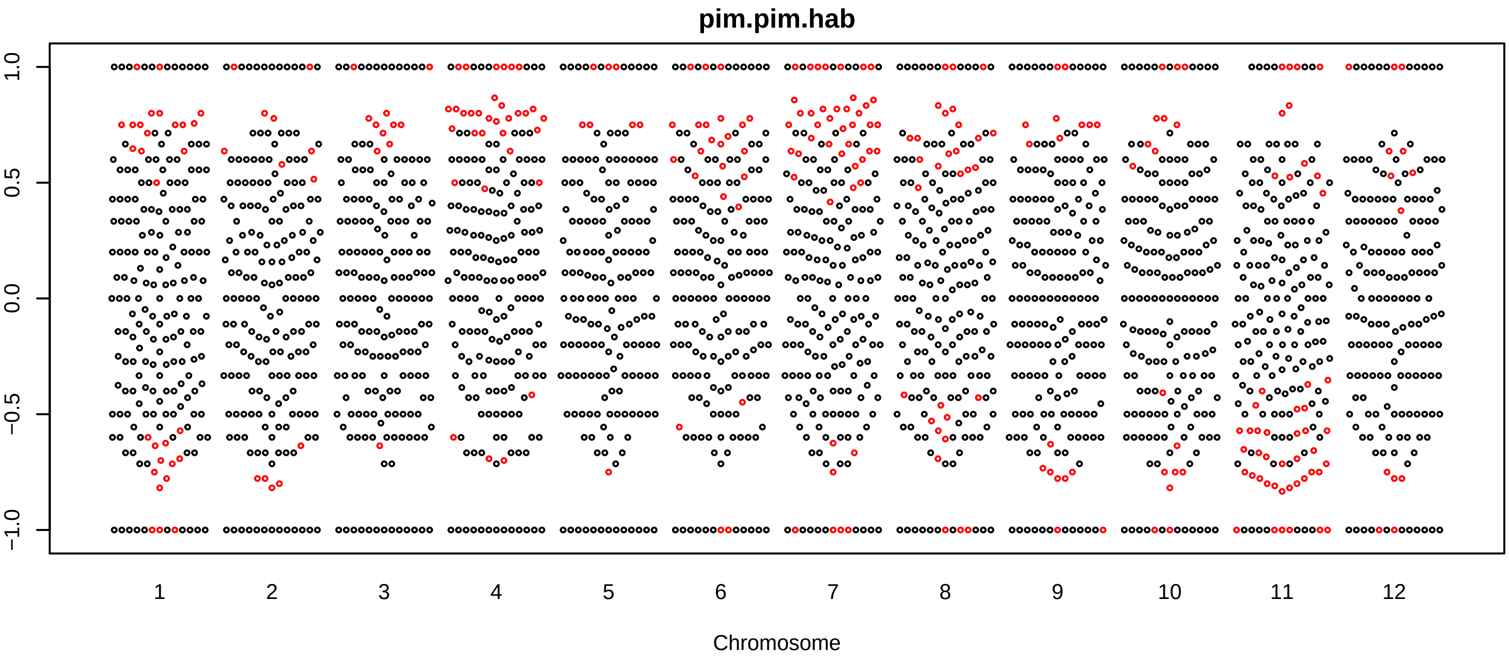
pim.pim.hab
1.0
0.5
0.0
−0.5
−1.0
1 2 3 4 5 6 7 8 9 10 11 12
Chromosome

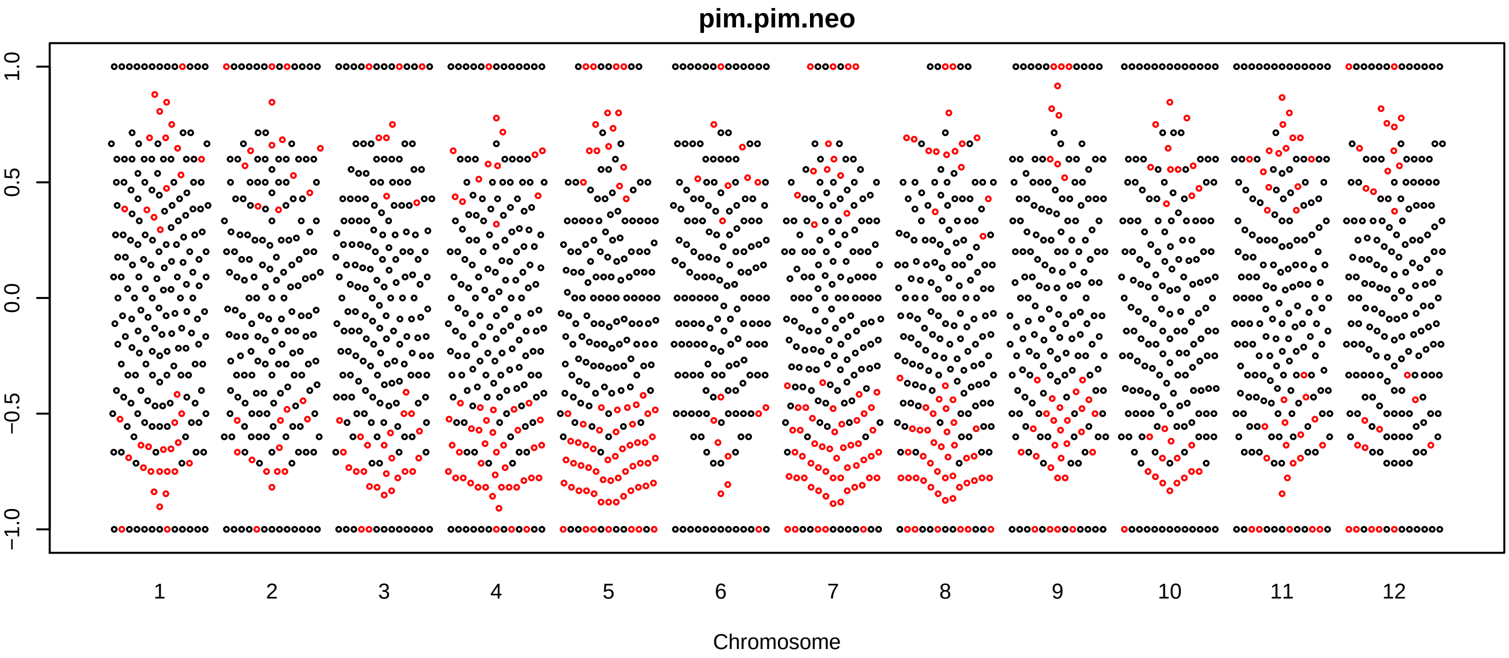
pim.pim.neo
1.0
0.5
0.0
−0.5
−1.0
1 2 3 4 5 6 7 8 9 10 11 12
Chromosome

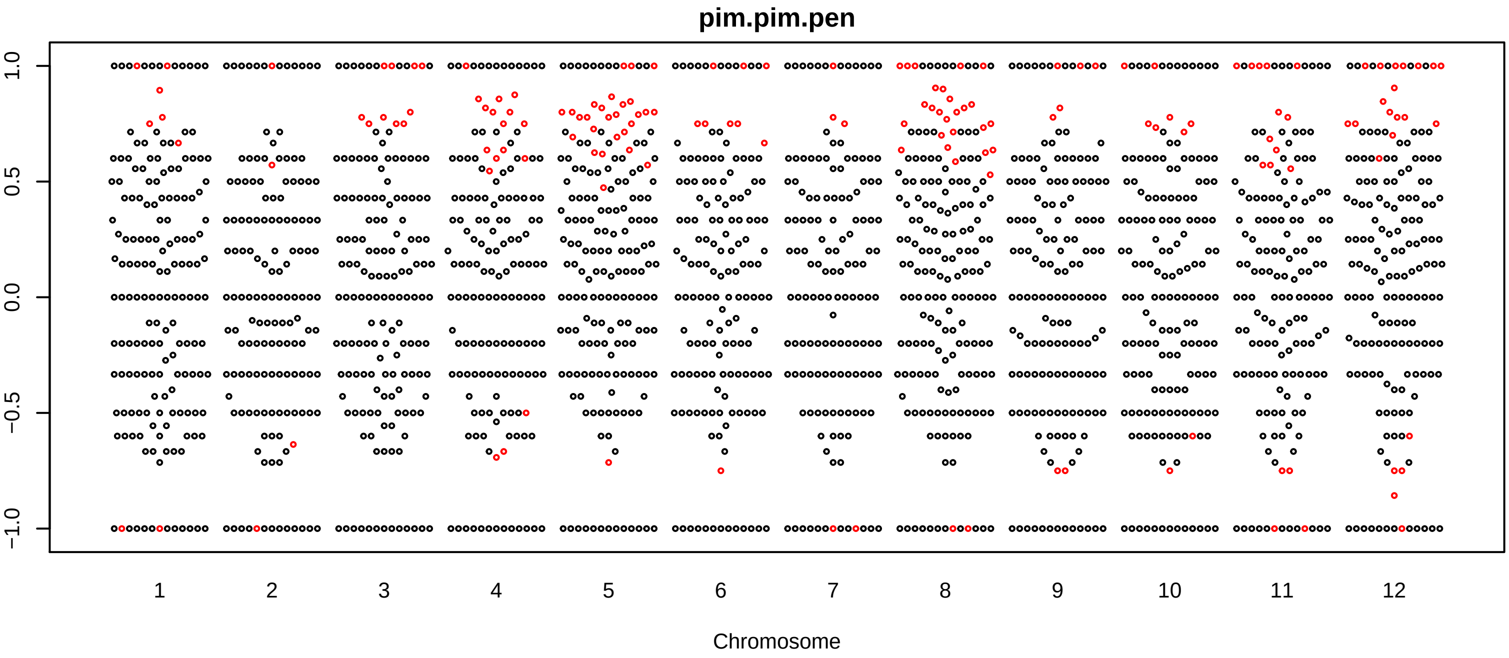
pim.pim.pen
1.0
0.5
0.0
−0.5
−1.0
1 2 3 4 5 6 7 8 9 10 11 12
Chromosome

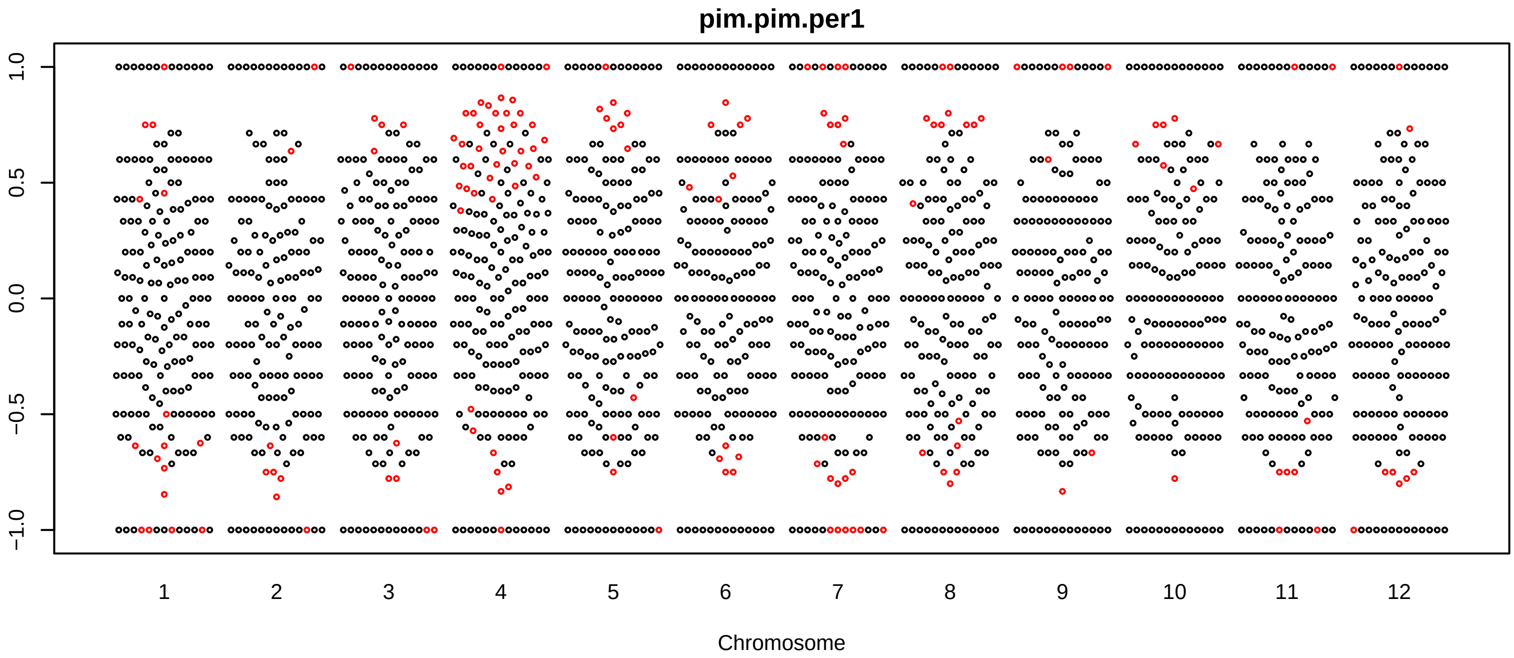
pim.pim.per1
1.0
0.5
0.0
−0.5
−1.0
1
2
3
4
5
6
7
8
9
10
11
12
Chromosome

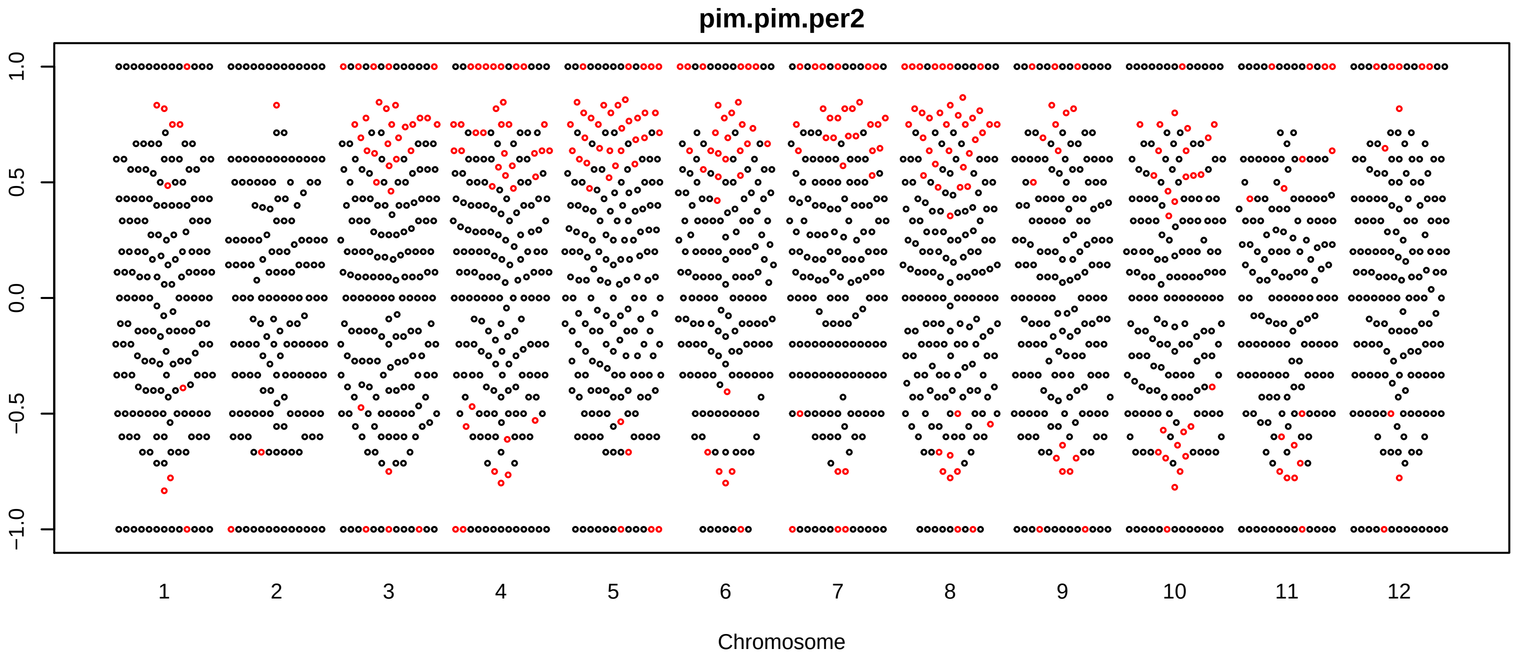
pim.pim.per2
1.0
0.5
0.0
−0.5
−1.0
1
2
3
4
5
6
7
8
9
10
11
12
Chromosome
