# Supplementary material for: Assessing biological factors affecting postspeciation introgression
Source: Evol Lett. 2020 Feb 28;4(2):137–54. doi: 10.1002/evl3.159 (PMC7156103; doi:10.1002/evl3.159)
Supplement: Supplementary file 4 — Figure S4. The distribution of inferred tree topologies across the genome, for each trio in our mating system tests. [file EVL3-4-137-s004.pdf]

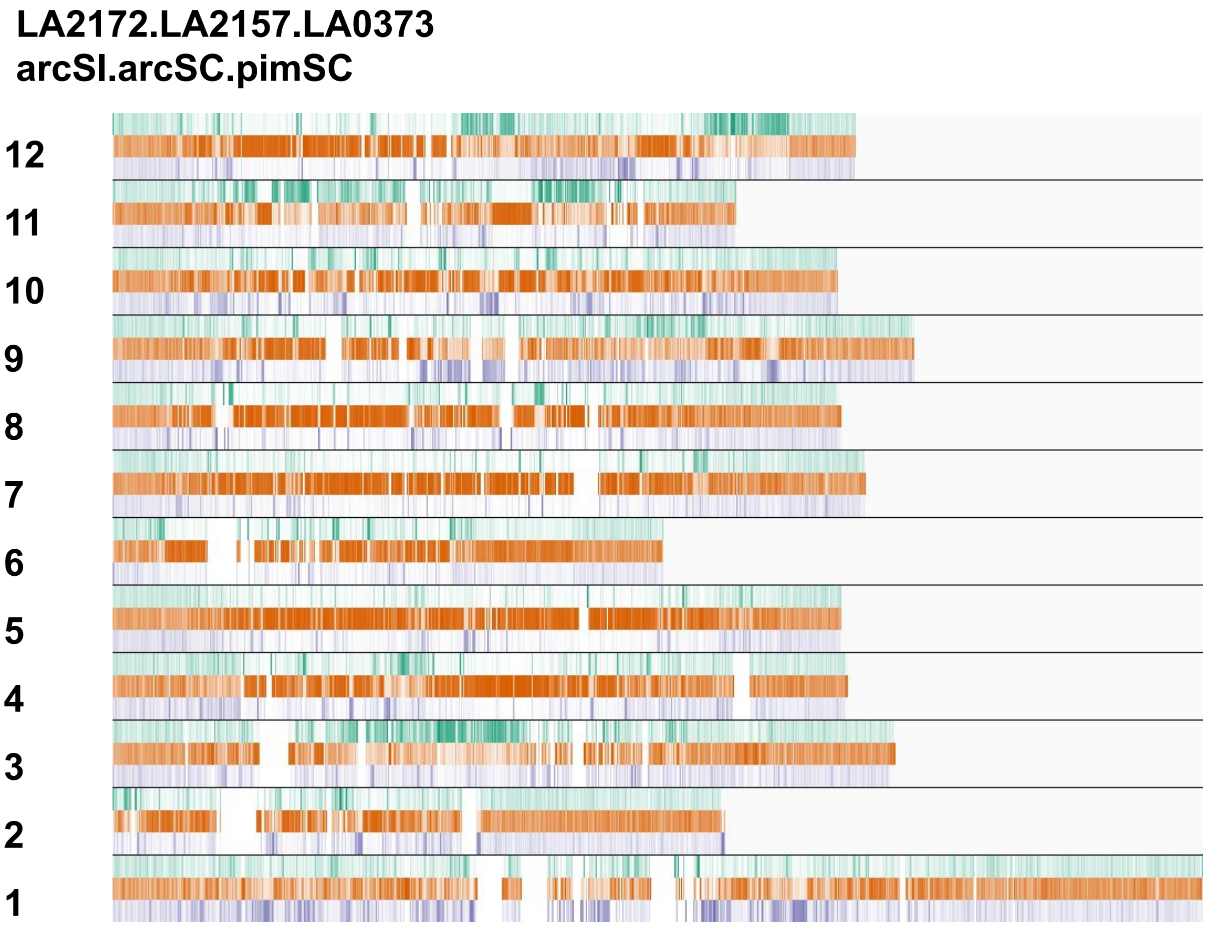
LA2172.LA2157.LA0373
arcSI.arcSC.pimSC
12
11
10
9
8
7
6
5
4
3
2
1

LA2172.LA2157.LA0400
arcSl.arcSC.pimSCv2

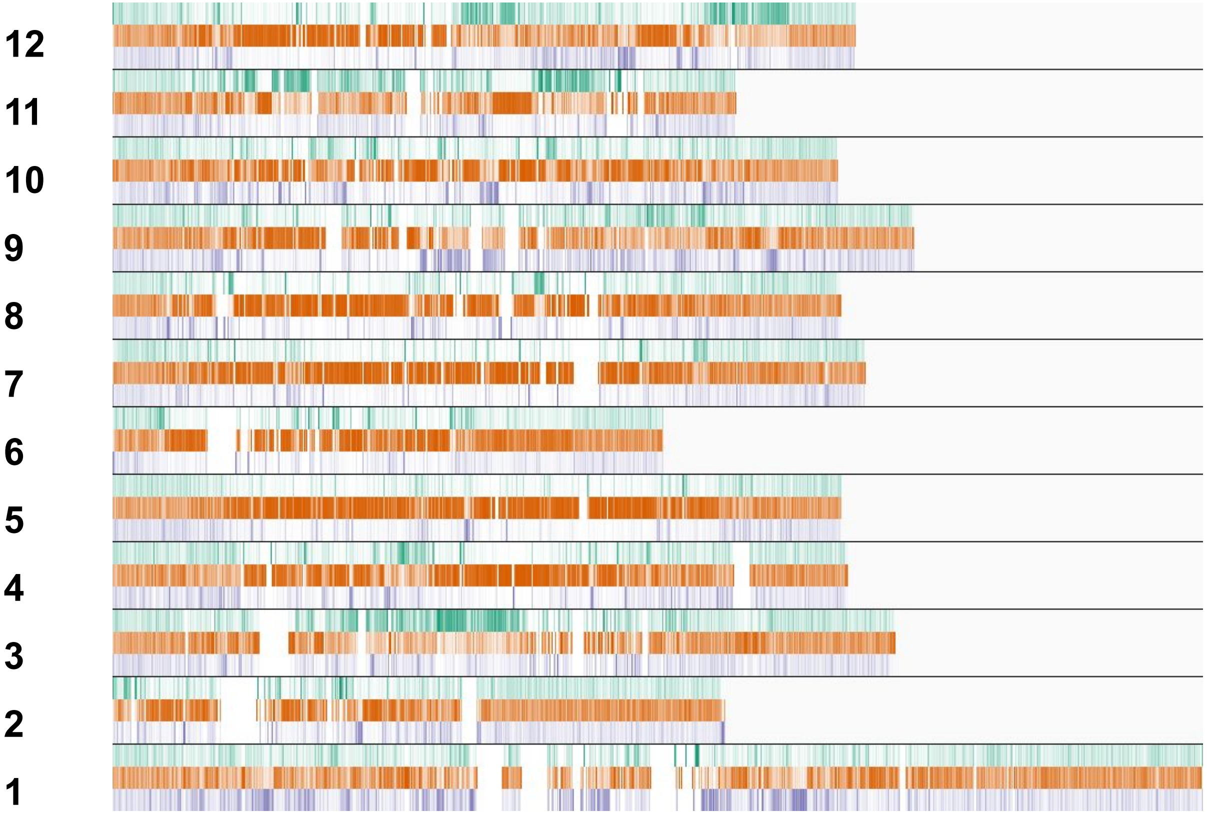

LA1278.PI128650.LA0373
perSI.perSC.pim.SC

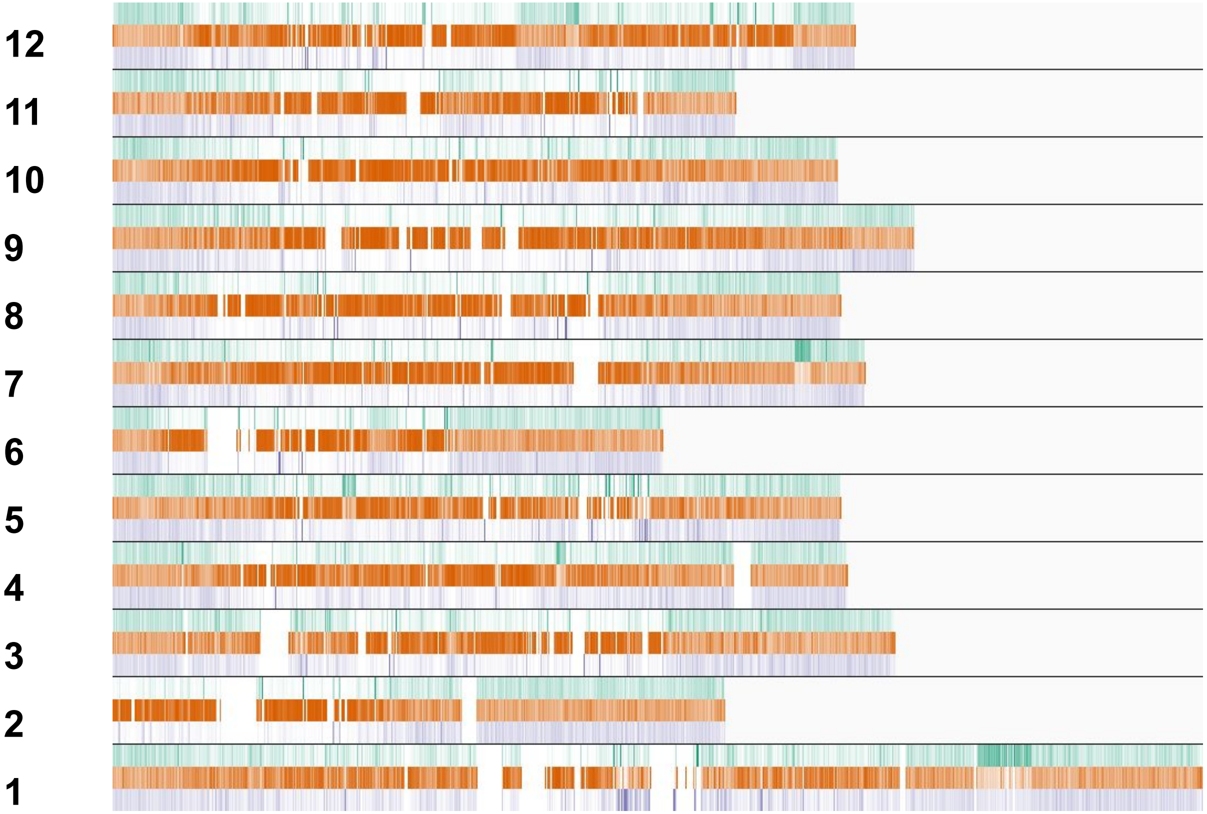

LA1278.PI128650.LA0400
perSl.perSC.pim.SC2

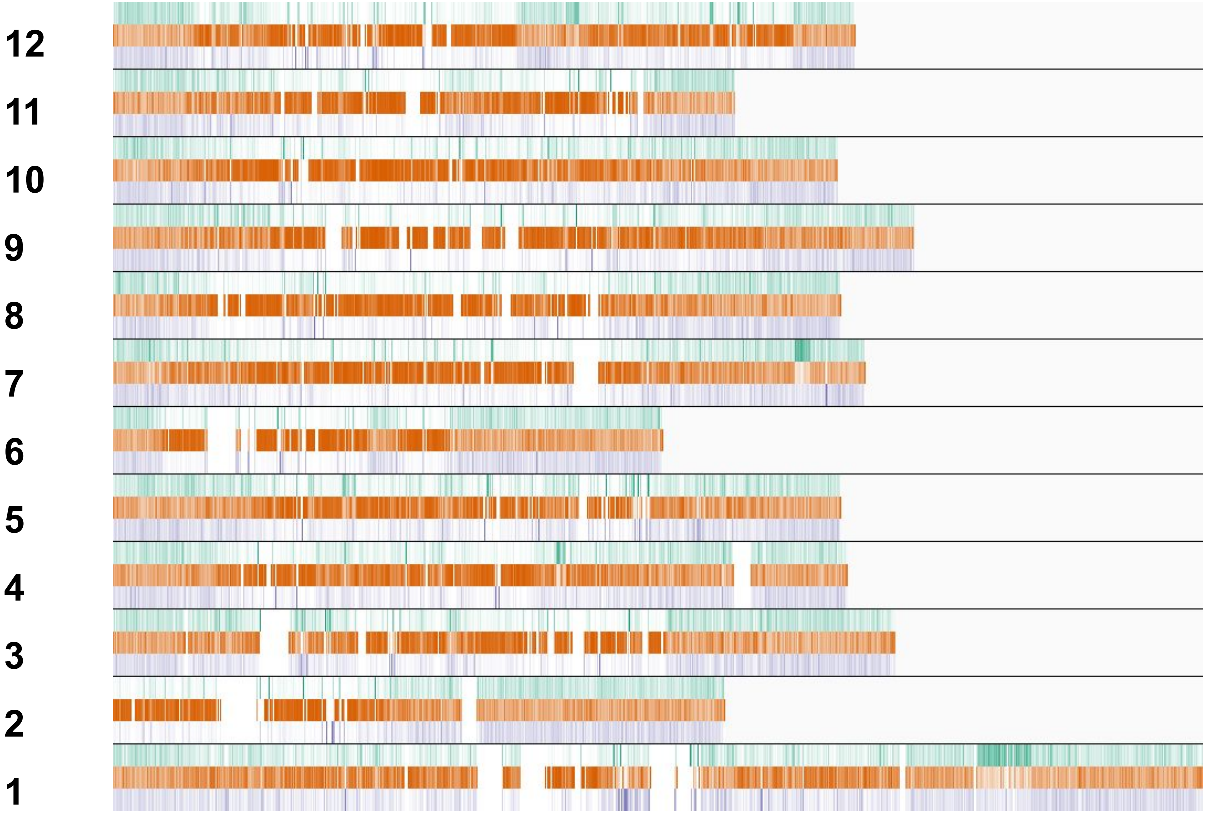

LA2157.LA2172.LA1718
arcSC.arcSI.habSI

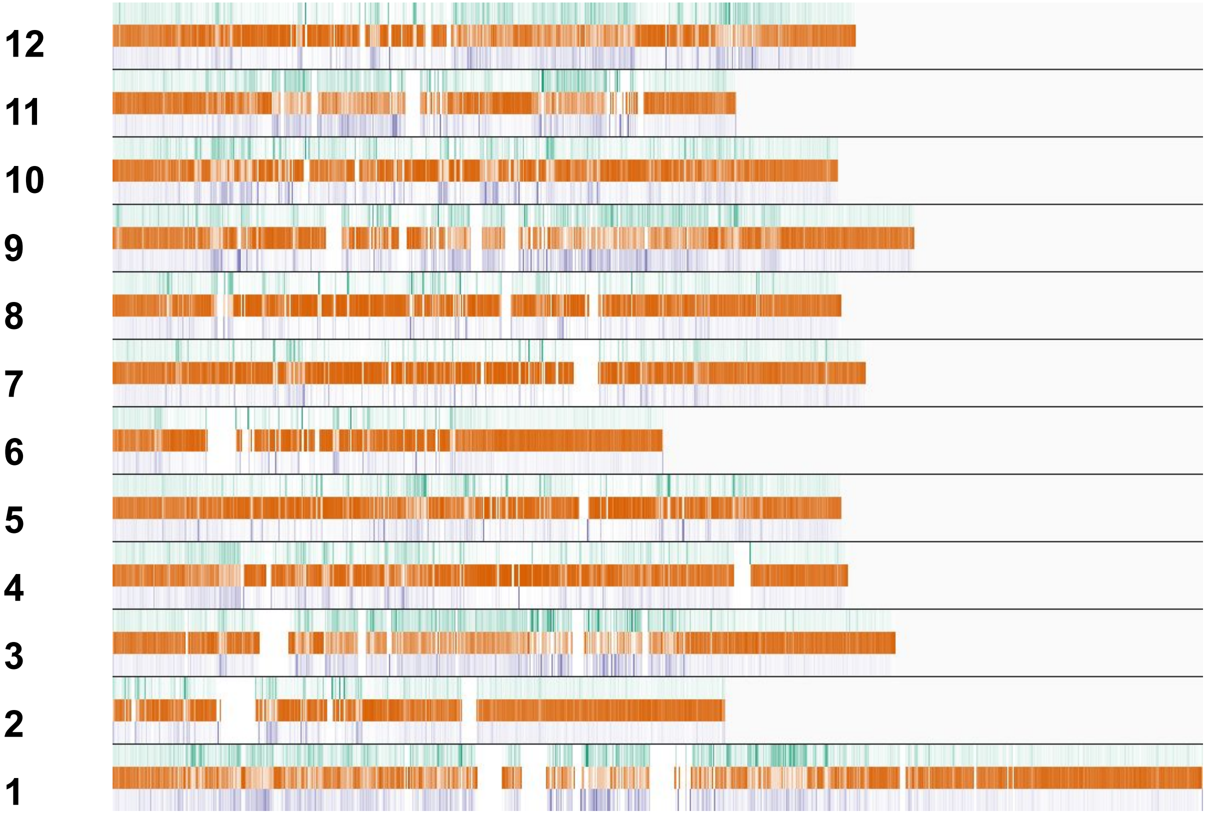

LA0407.LA1777.LA0118
habSC.habSI.corSI

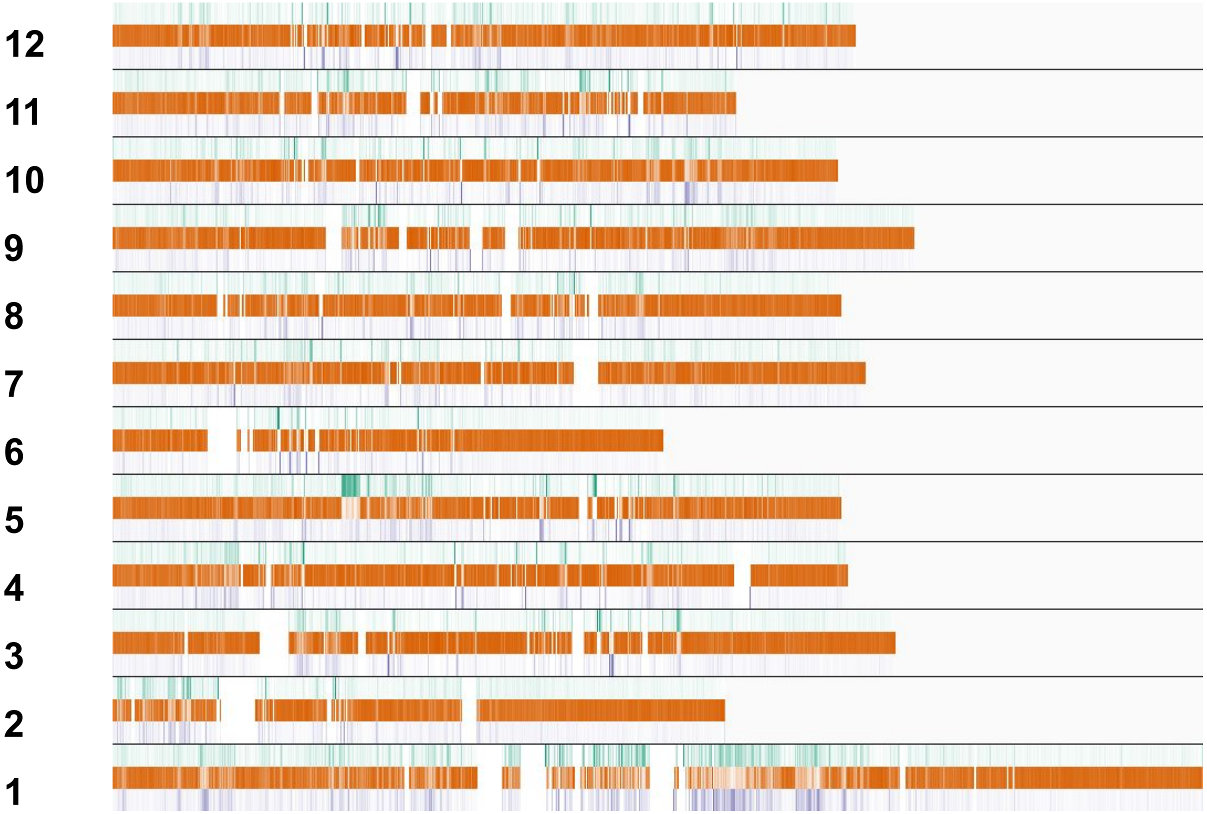

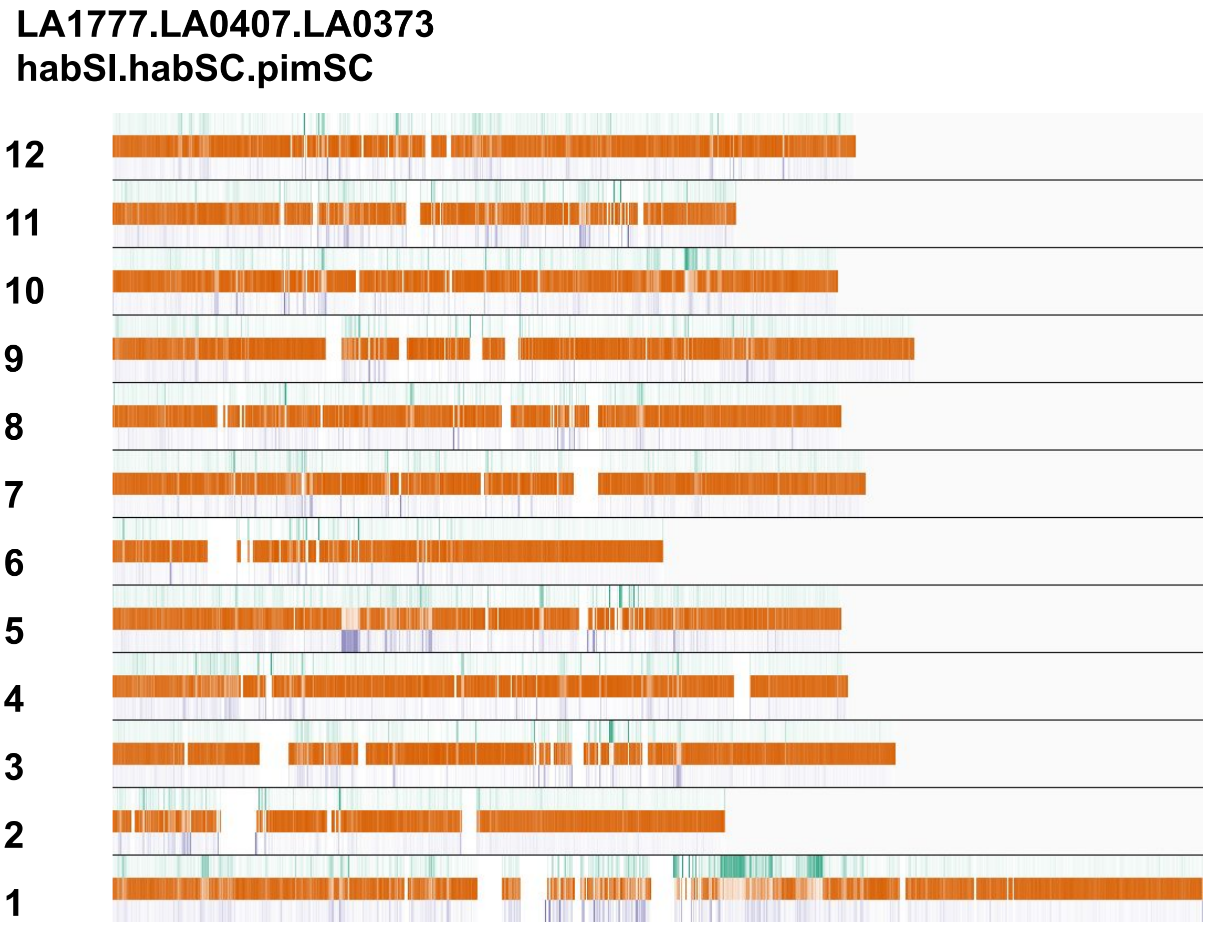
LA1777.LA0407.LA0373
habSI.habSC.pimSC
12
11
10
9
8
7
6
5
4
3
2
1

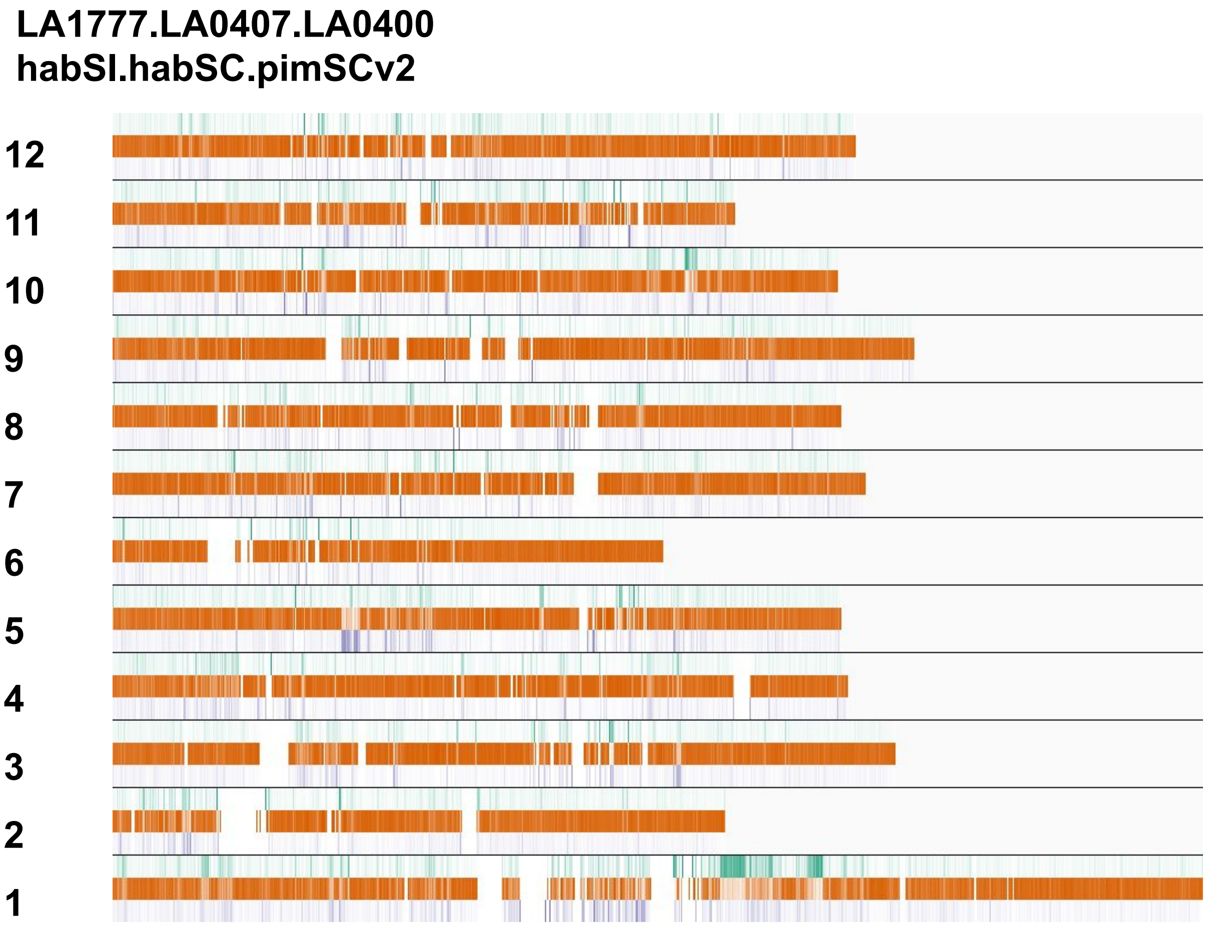
LA1777.LA0407.LA0400
habSI.habSC.pimSCv2
12
11
10
9
8
7
6
5
4
3
2
1
