# Supplementary material for: Assessing biological factors affecting postspeciation introgression
Source: Evol Lett. 2020 Feb 28;4(2):137–54. doi: 10.1002/evl3.159 (PMC7156103; doi:10.1002/evl3.159)

LA1044.LA0483.LA0746
gal.gal.che

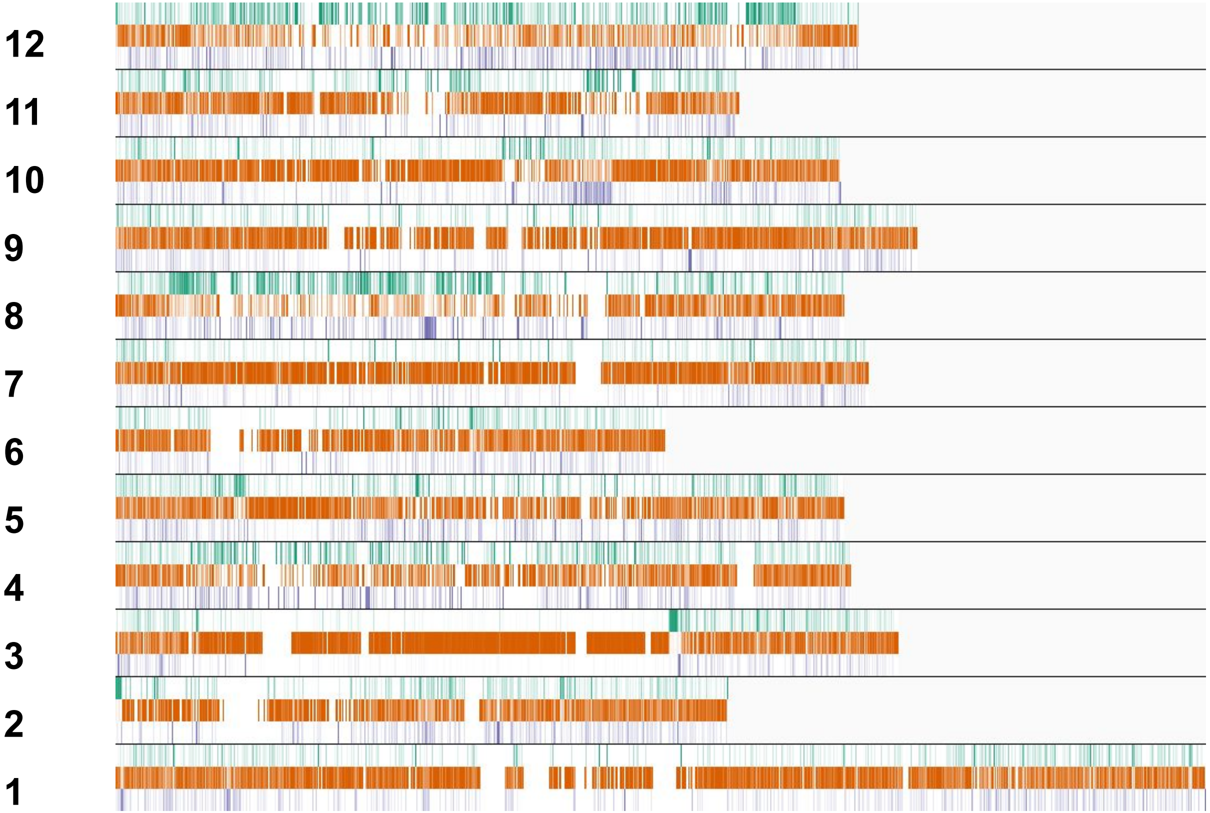

LA2172.LA2157.LA2147
arc.arc.pim

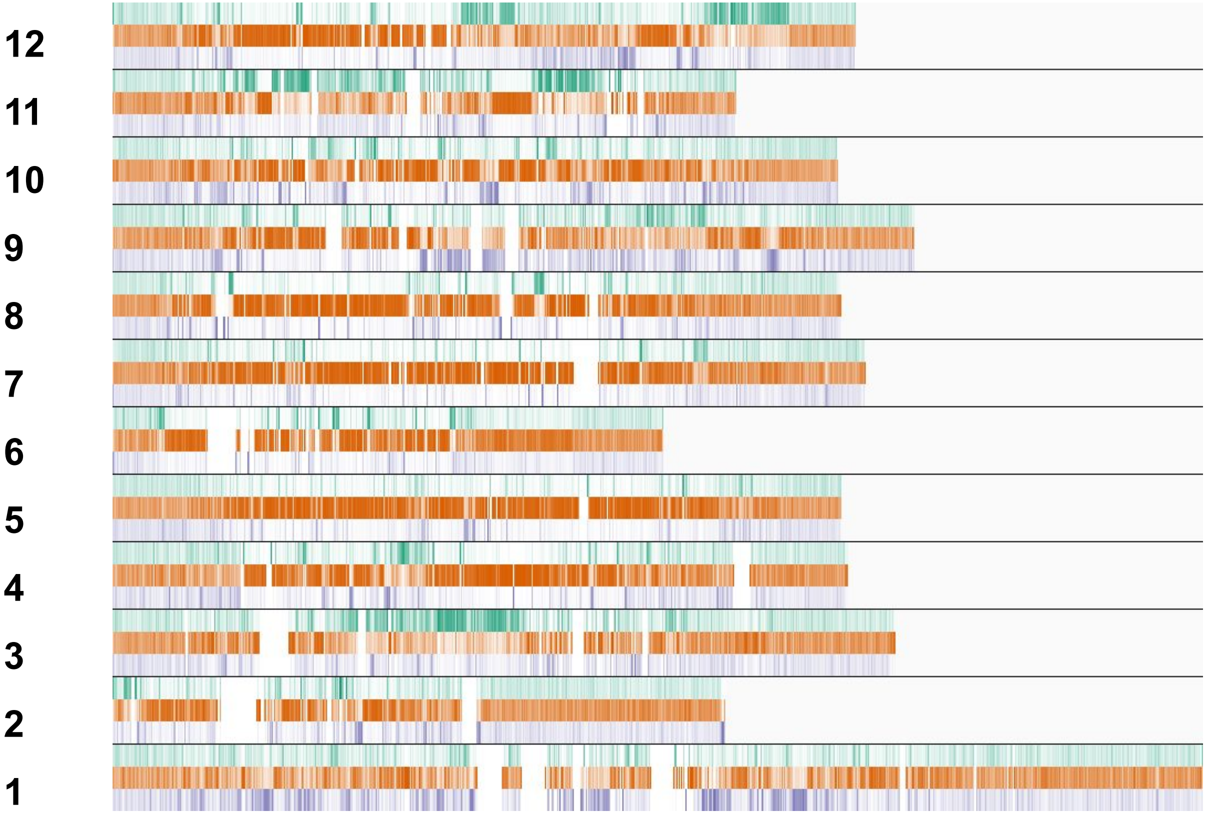

LA1375.LA1246.LA2133
pim.pim.neo

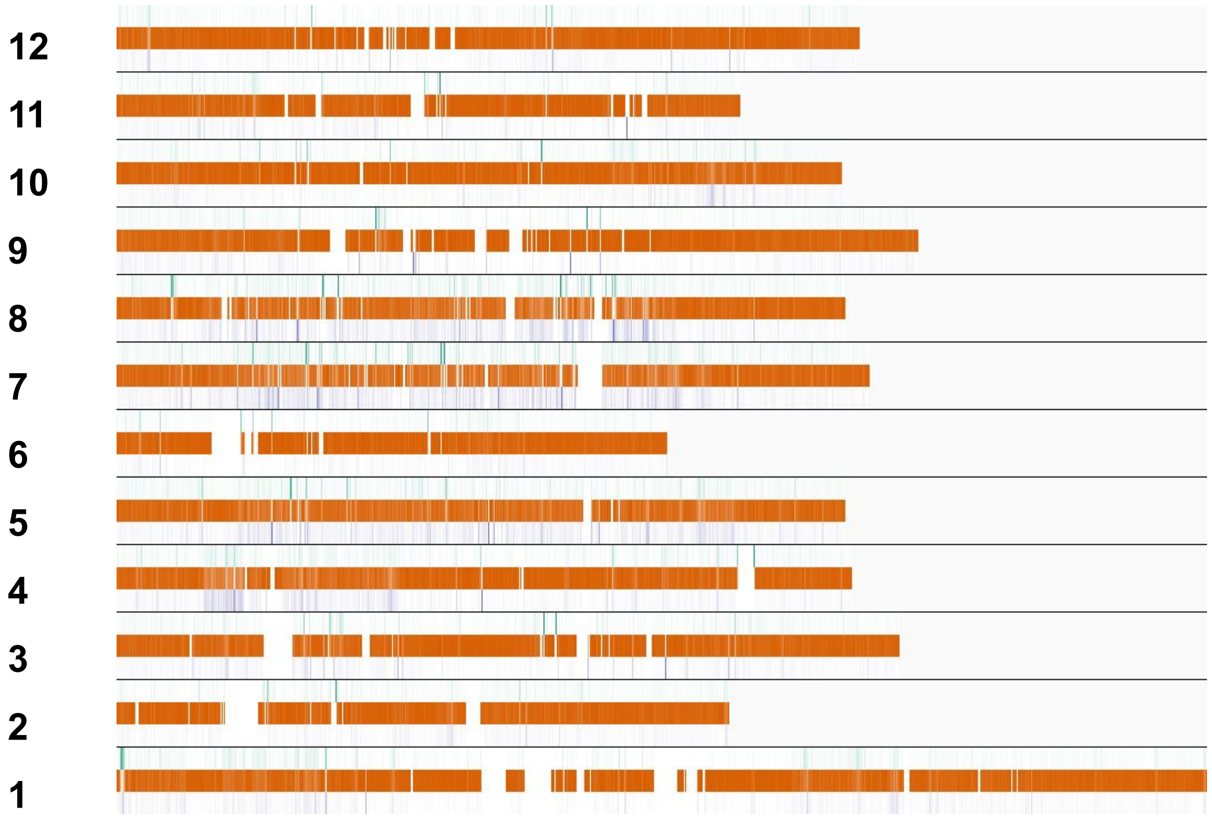

LA1582.LA1933.LA1969
pim.pim.chi

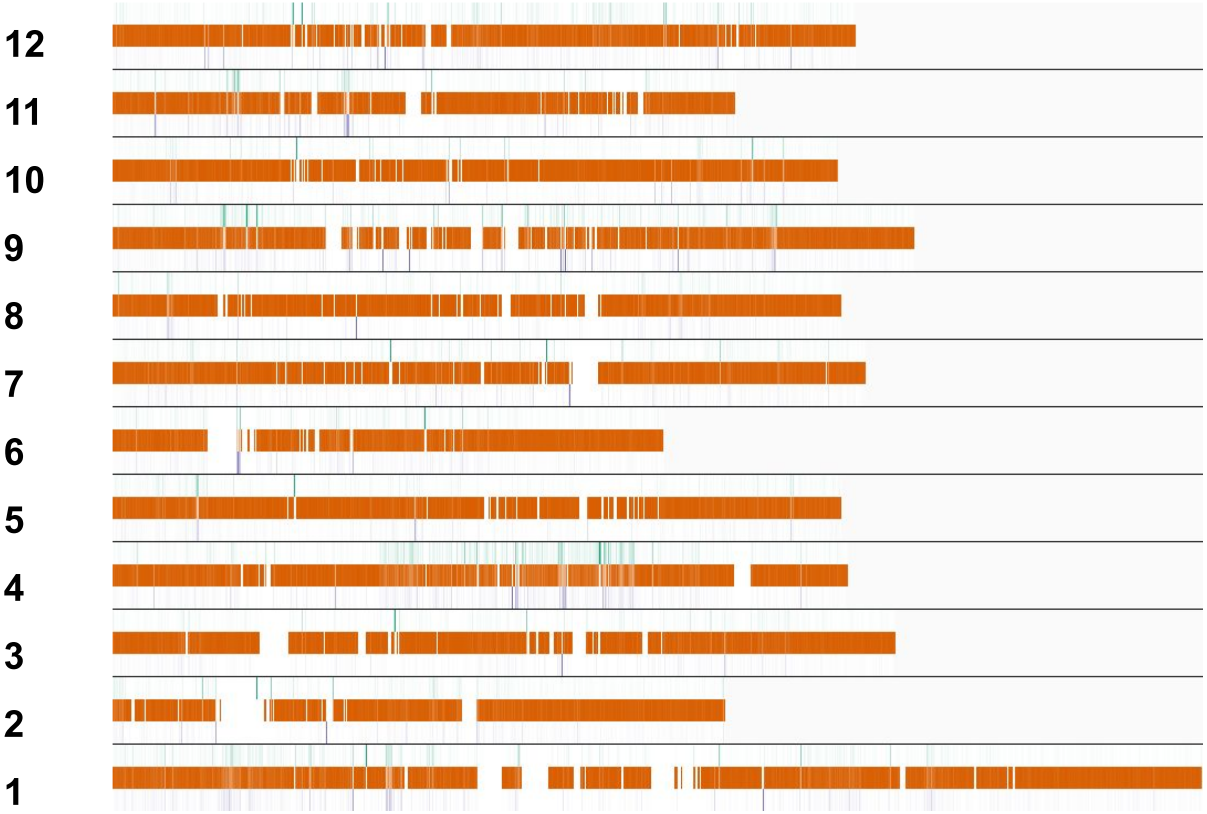

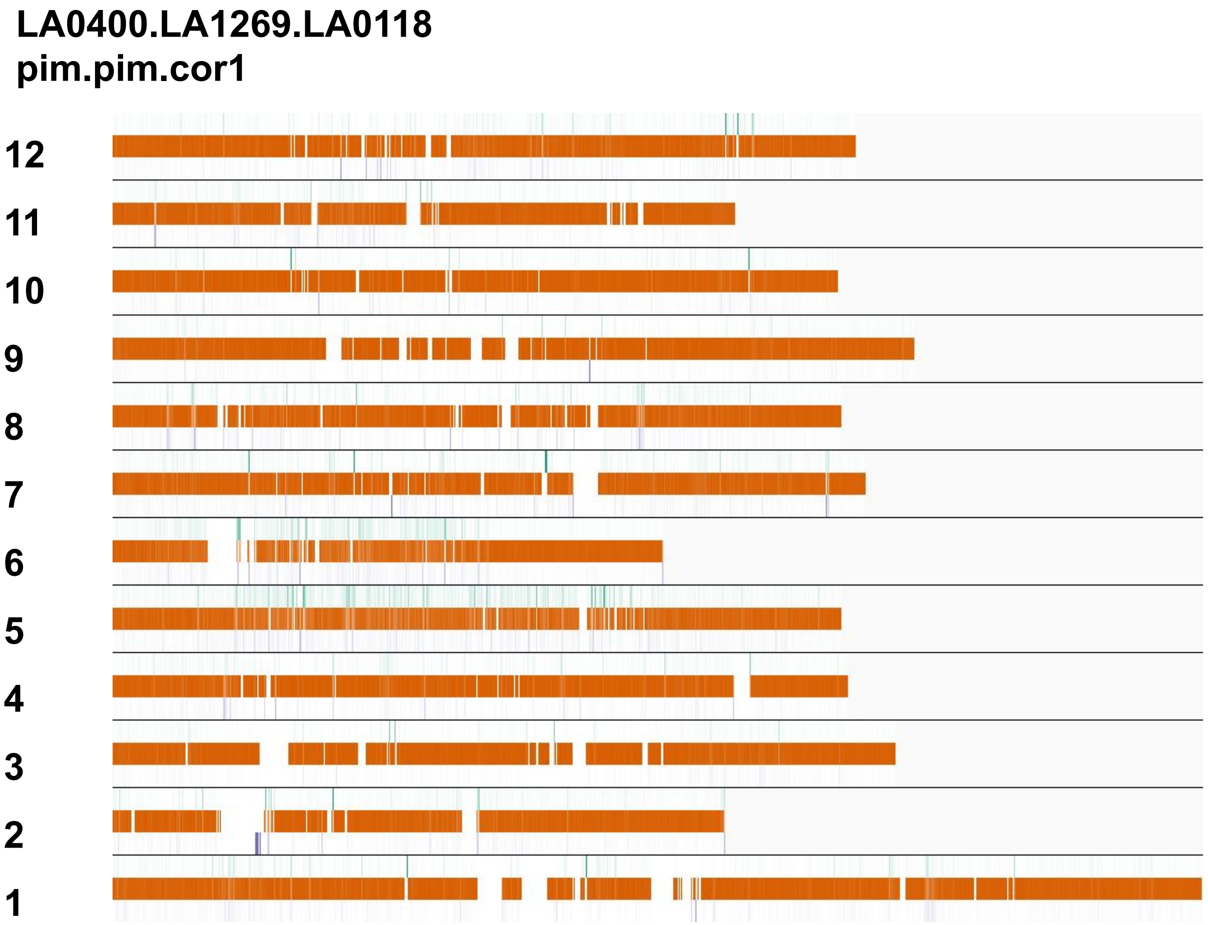
LA0400.LA1269.LA0118
pim.pim.cor1
12
11
10
9
8
7
6
5
4
3
2
1

LA1617.LA1521.LA0118
pim.pim.cor2

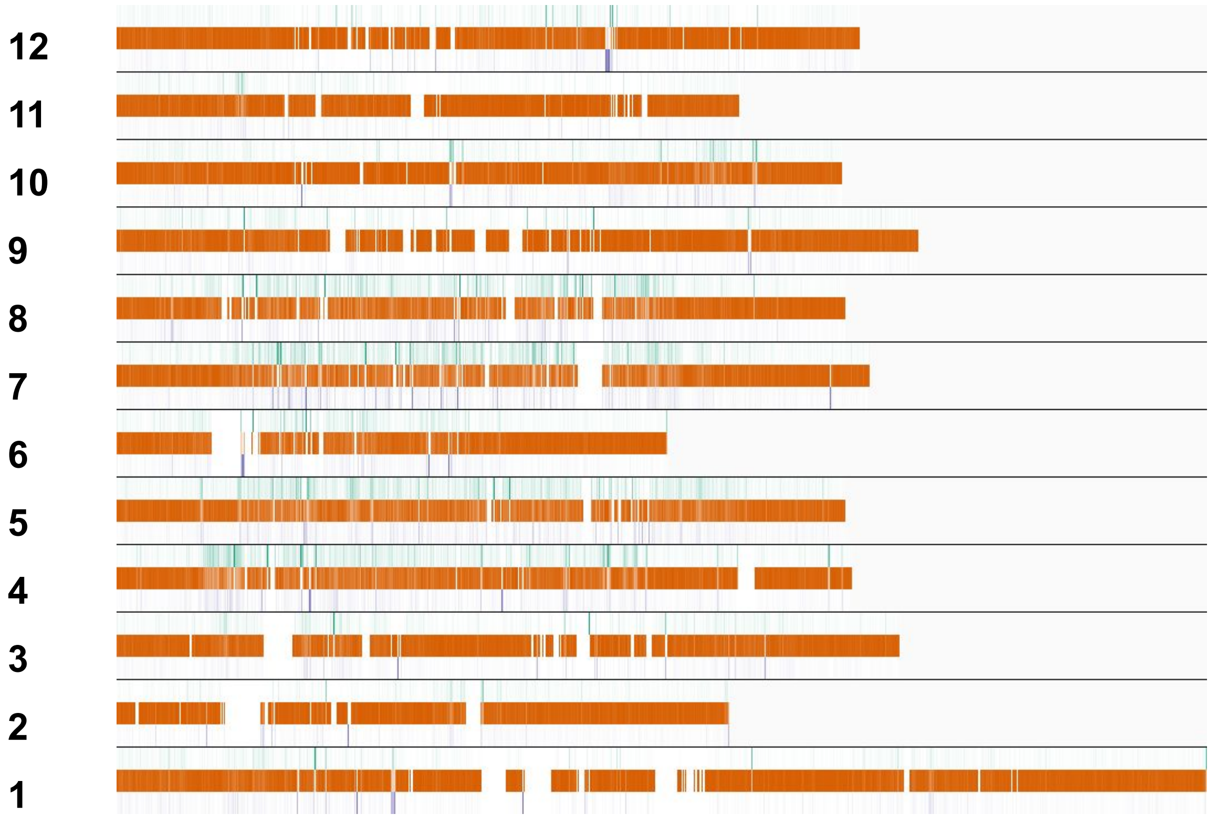

LA1595.LA1341.LA1278
pim.pim.per1

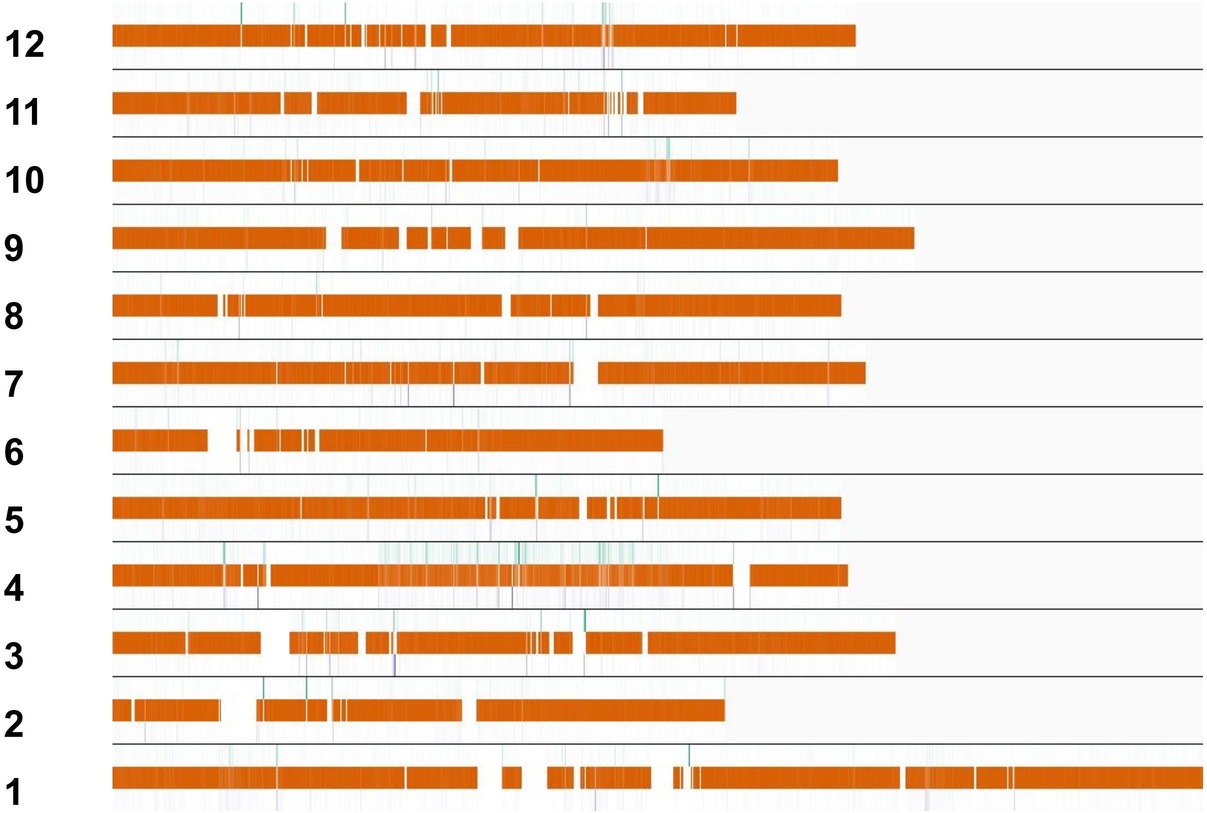

LA1617.LA1269.LA1278
pim.pim.per2

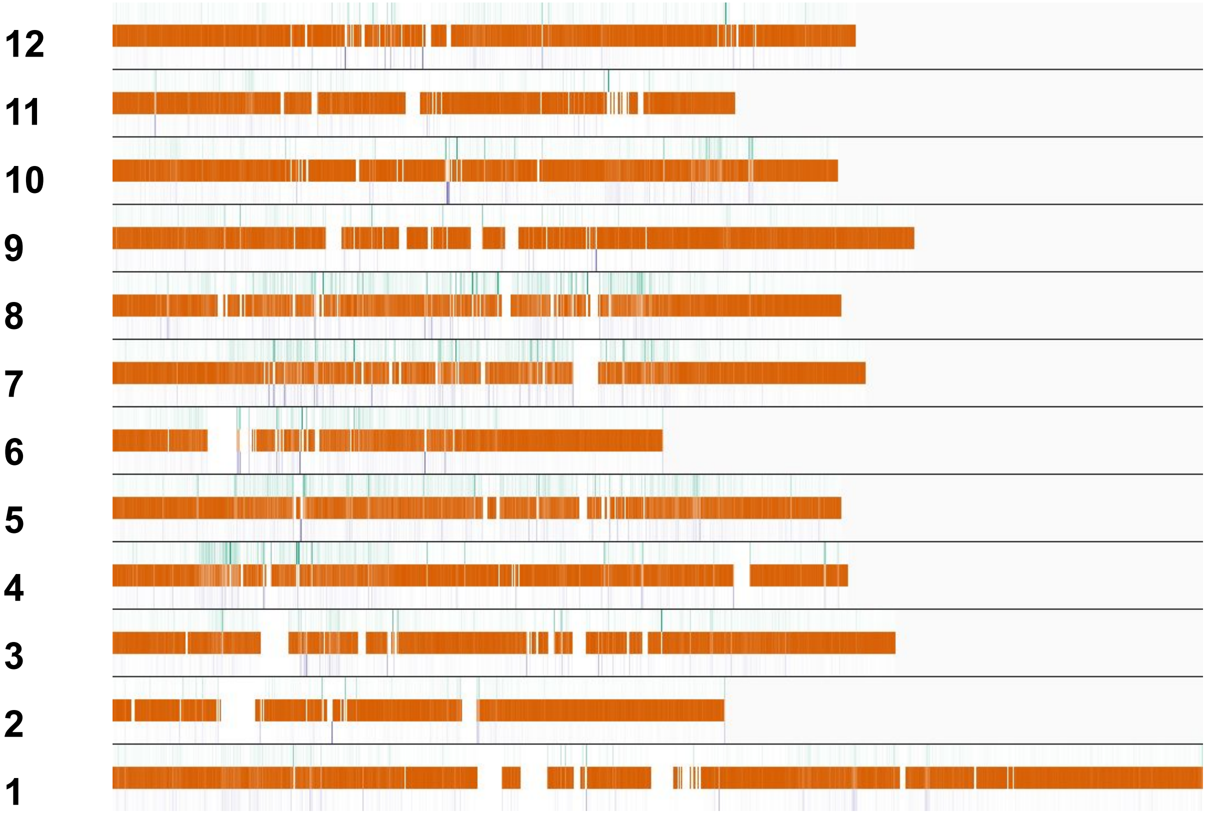

LA0417.LA0442.LA1777
pim.pim.hab

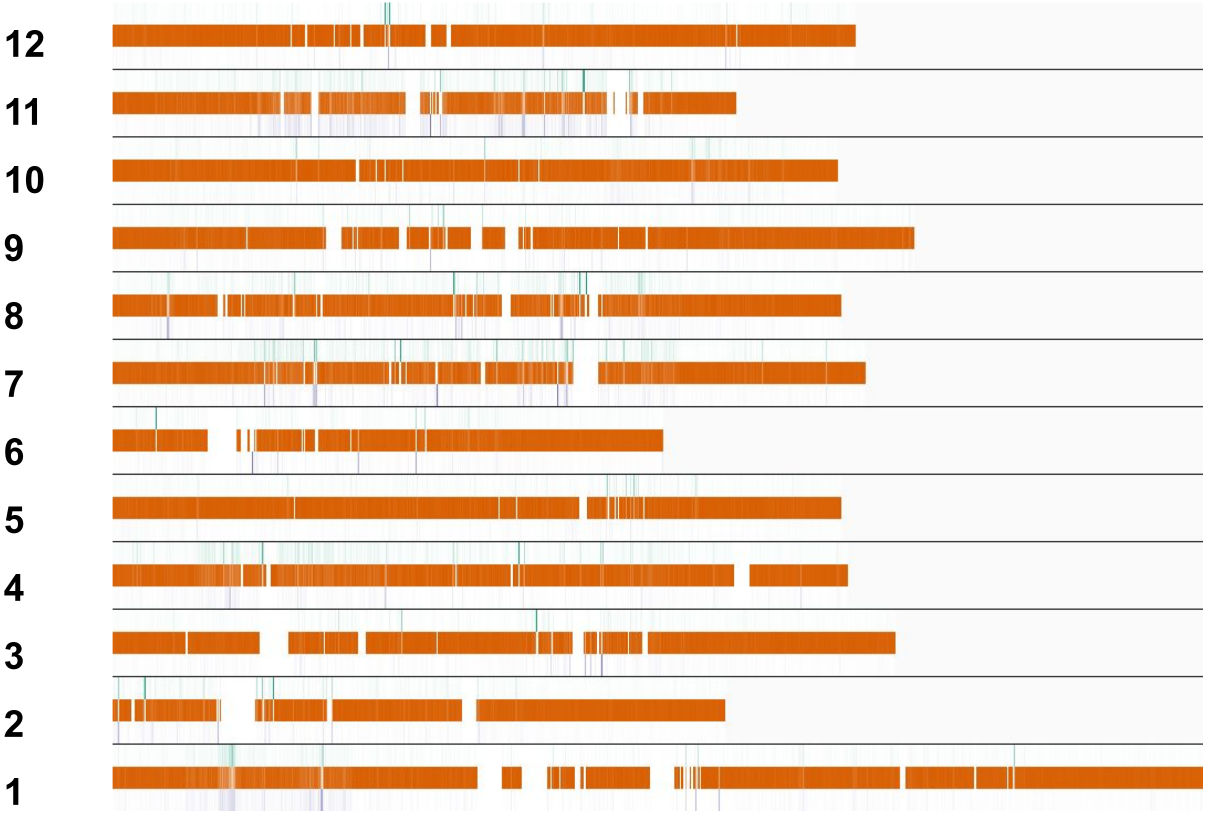

LA1245.LA1269.LA1272
pim.pim.pen

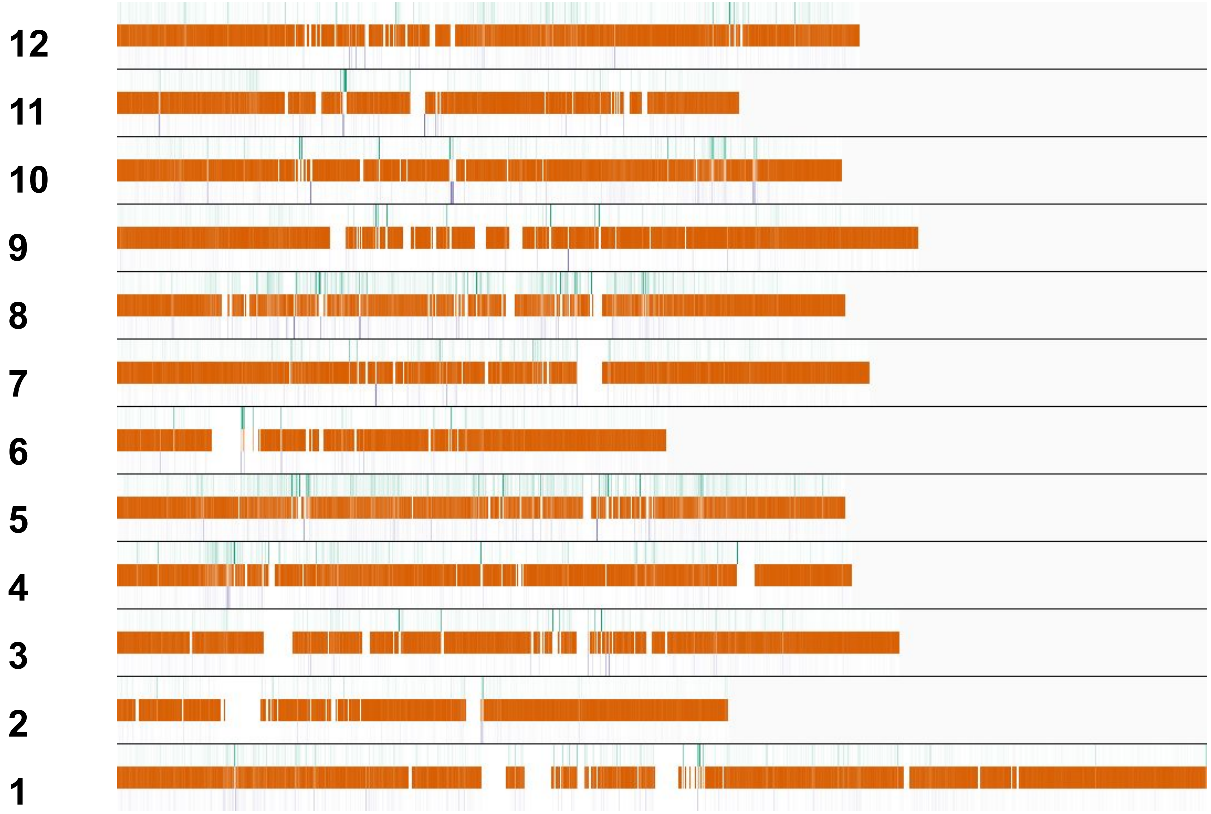

LA2172.LA2157.LA1718
arc.arc.hab

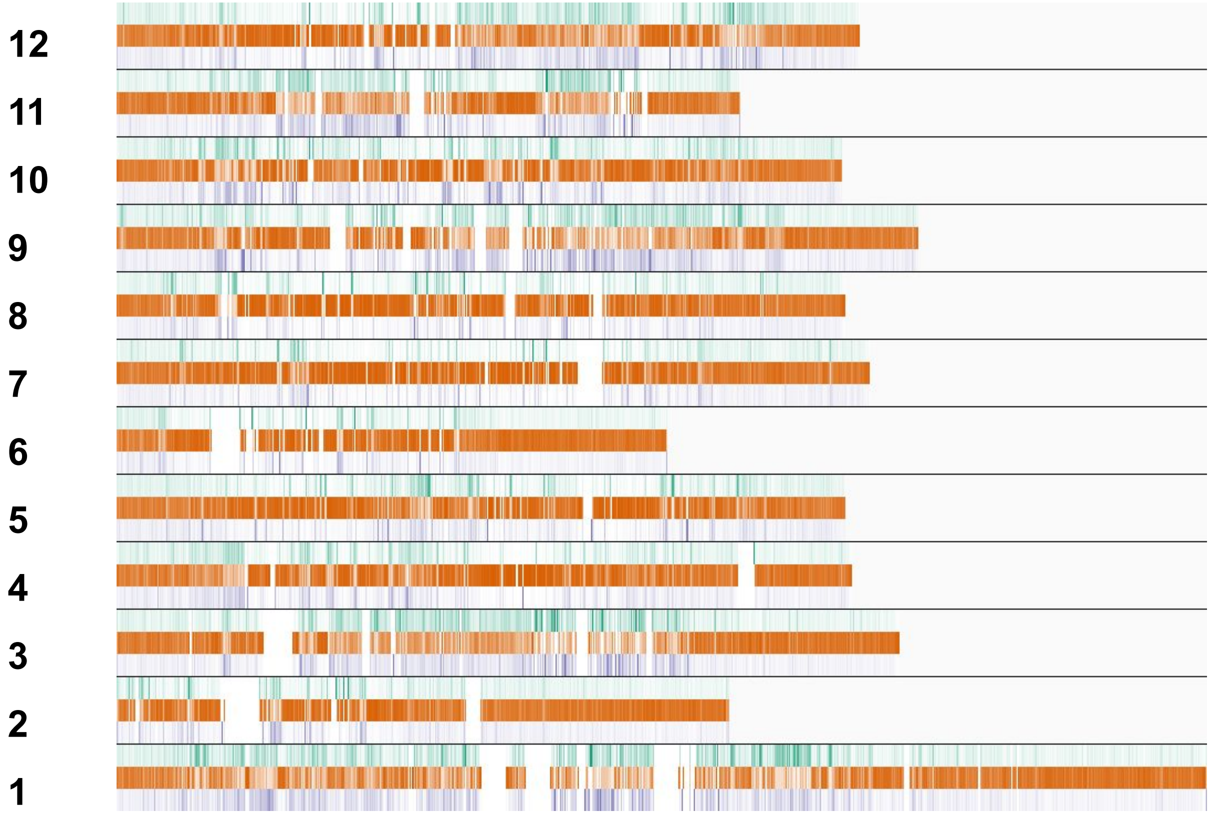

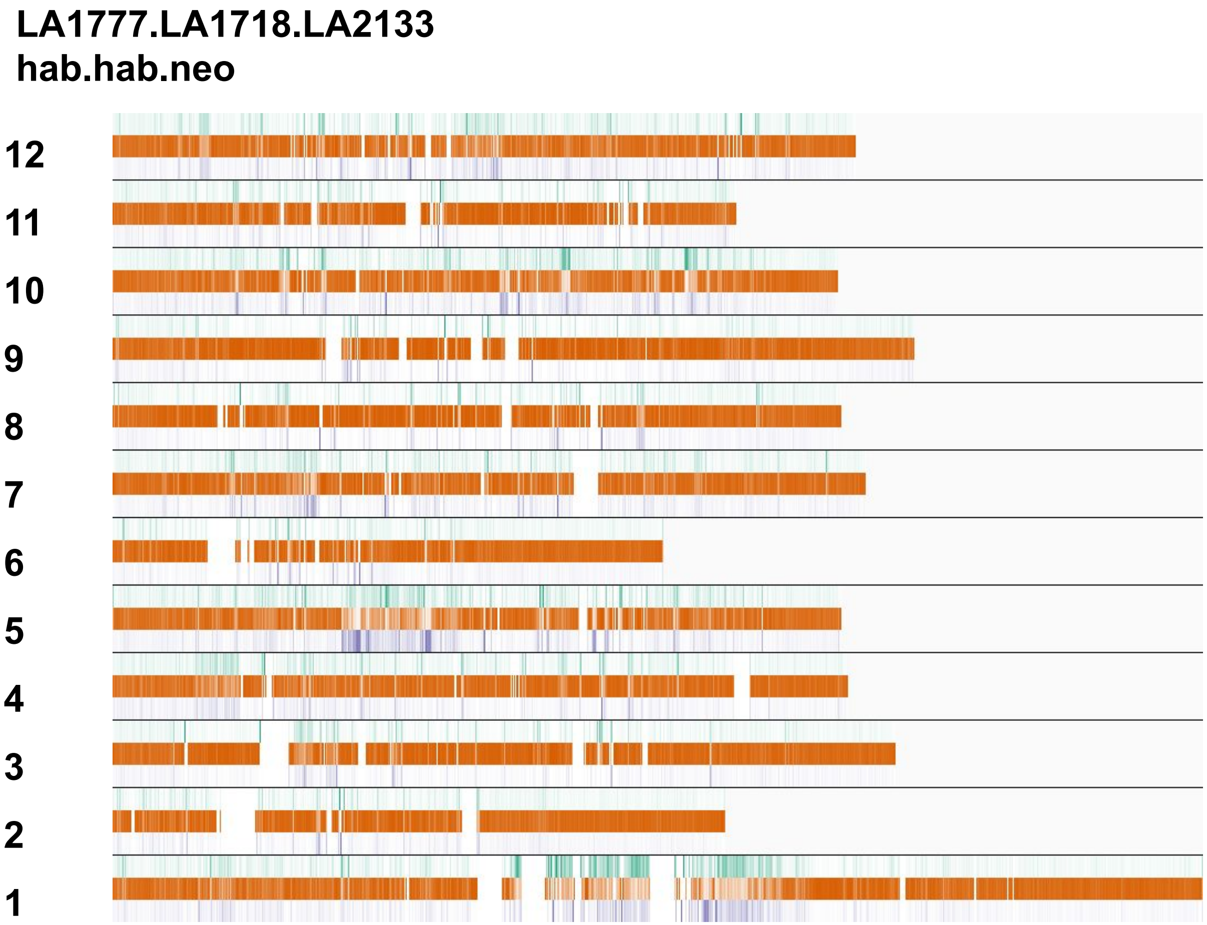
LA1777.LA1718.LA2133
hab.hab.neo
12
11
10
9
8
7
6
5
4
3
2
1

LA0407.LA1777.LA0118
hab.hab.cor

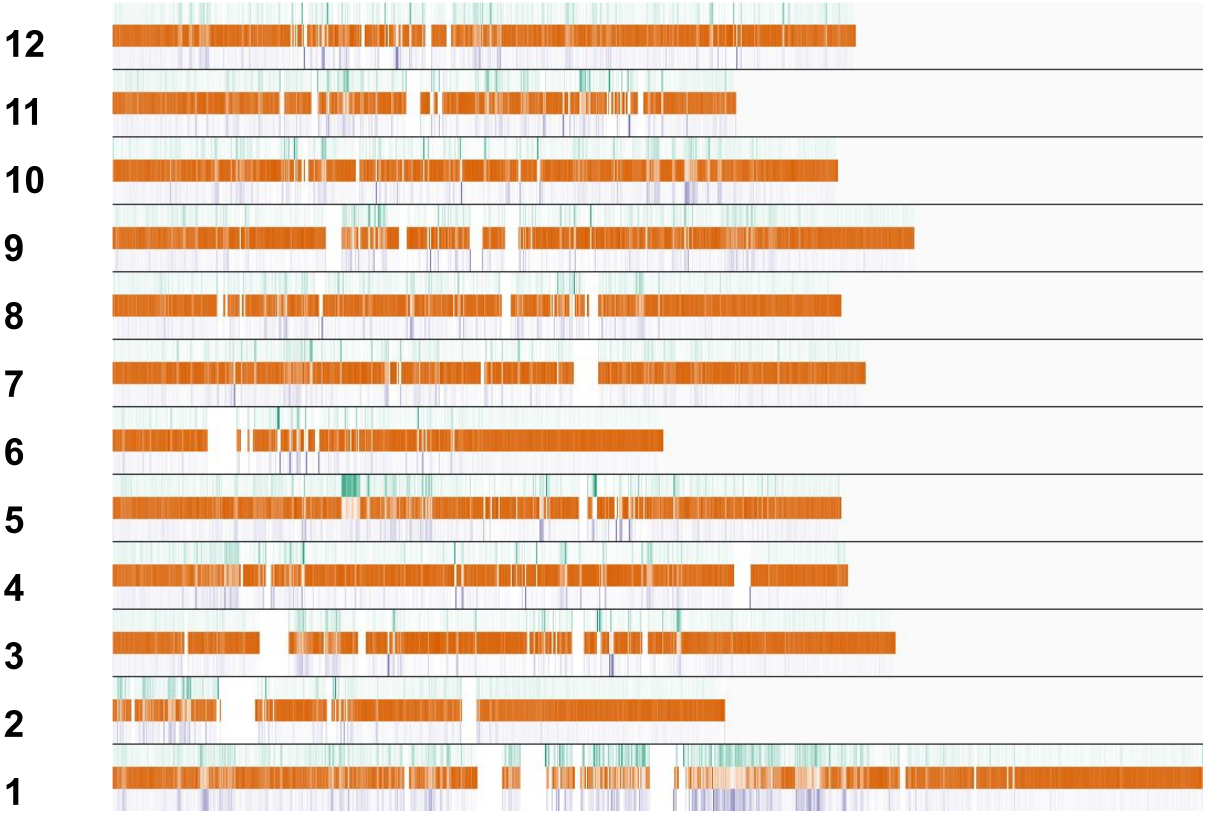

LA1983.LA1365.LA1718
hua.hua.hab

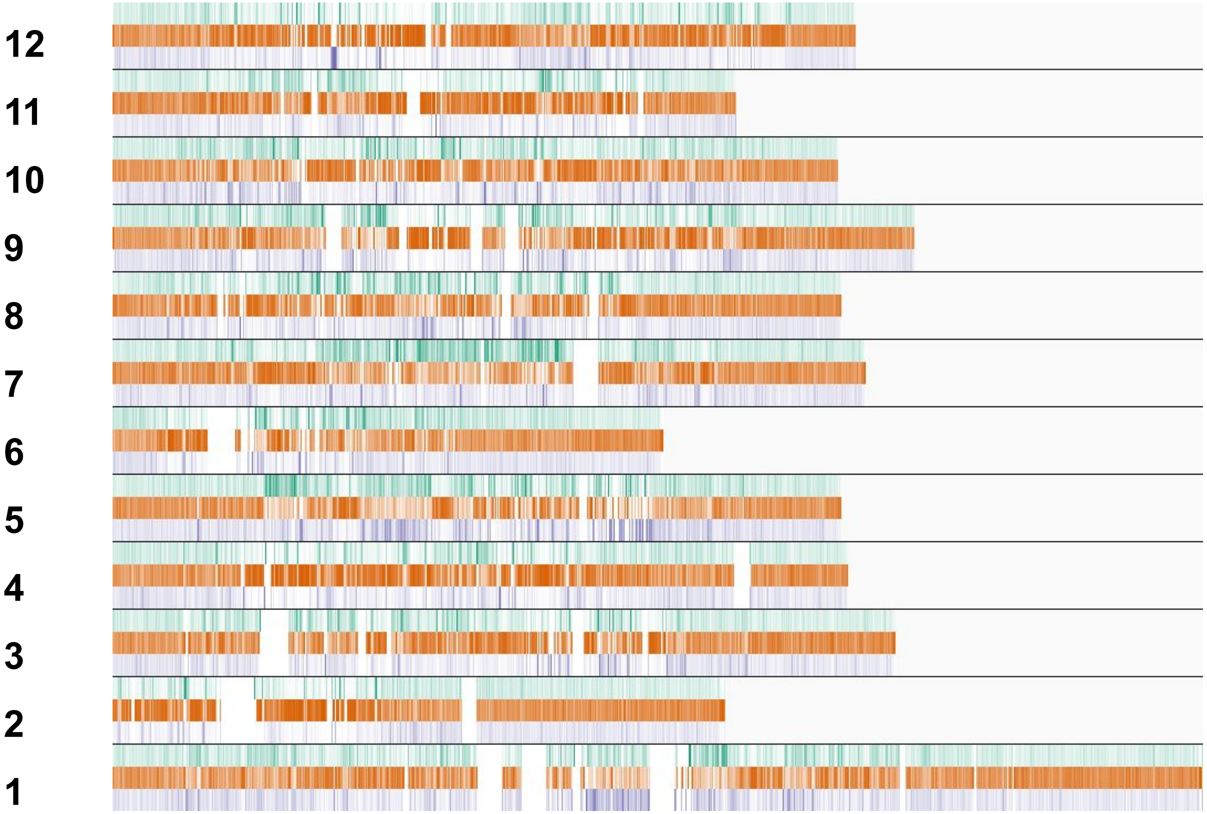

Supplement: Supplementary file 5 — Figure S5. The distribution of inferred tree topologies across the genome for each trio in our geographic tests. [file EVL3-4-137-s005.pdf]
