# Supplementary material for: Assessing biological factors affecting postspeciation introgression
Source: Evol Lett. 2020 Feb 28;4(2):137–54. doi: 10.1002/evl3.159 (PMC7156103; doi:10.1002/evl3.159)
Supplement: Supplementary file 7 [file EVL3-4-137-s007.docx]

**Supplemental Methods and Results**

*Mapping raw reads to reference genome*

To combine the data from three different genome sequencing projects, we trimmed and re-mapped raw reads back to the reference genome of tomato, *S. lycopersicum* version 2.50 (The Tomato Genome Consortium, 2012). Trimmomatic was first used for removing adaptors and quality trimming, using a 4-base sliding window, and cutting when the average quality per base dropped below 20 and/or when reads were less than 36 bp long (Bolger et al., 2014). All retained reads were then mapped back to the reference genome using bwa mem (Li, 2013), sorted and indexed with samtools (Li et al., 2009), and *mpileup* was used for generating a pileup file. bcftools call generated a BCF file for each of the 12 chromosomes (to facilitate downstream analyses) for all accessions. We then filtered the data using varFilter, part of vcfutils.pl, such that the minimum base quality was required to be > 30 and read depth >10 generating a filtered VCF file for each chromosome. All base calls that did not pass quality filtering were then masked and converted to mvf format using MVFtools (Pease and Rosenzweig, 2018) prior to calculating introgression statistics. For each 100kb window, a z-score was calculated to determine if the D value in this specific window was different from zero; individual 100kb windows with D significantly different than 0 are shown as colored points while non-significant windows are represented as black (Supplemental Figure 3) or grey (Figure 3 and 4) circles.

*Demographic parameters for D_p_ analysis*

With respect to specific parameters used in our simulations, the timing of the P1/P2 split and the P3/P1P2 split were chosen to be sufficiently close in time for there to be incomplete lineage sorting in the dataset (D­­_p_ tests against a background of ILS). Because there are many possible times that could generate ILS, the split times were chosen to be with the plausible range of coalescence times estimated from empirical datasets, although within this the exact values used are arbitrary. Similarly, introgression needs to happen after the timing of the P1/P2 split, and we wanted to assess how the timing affected D­­_p_, but beyond that the choice of exact values was arbitrary. The outgroup split is much farther back to ensure that every simulated gene tree is rooted the same way. This is consistent with the empirical estimate for our dataset, where the split between potato (our outgroup) and the wild tomato clade is far older that the splits within this clade.

*Demographic and simulation parameters for D_2_ analysis*

To parameterize the null simulations of *D*_2_ for each trio, we compiled species-level demographic data from Pease *et al.* (2016), including estimates of heterozygosity and molecular clock estimates of speciation times (Supplemental Table 7a-c). Lineage-specific variation between P3 and P1/P2 accessions was specified in *ms* using the fold-difference in heterozygosity between species, which sometimes spanned several orders of magnitude. This is consistent with previous estimates of effective population size between species of *Solanum*, which can range from 1 x 10^4^ to 1 x 10^6^ (Roselius et al. 2005).

Note that our D2 simulations used windows in which we assumed no recombination. Although we have not evaluated this effect directly, our expectation is that the homogenizing effect of intralocus recombination within each window might make the test more conservative (less likely to reject the null hypothesis, which in this case is that the direction is C->B), under some specific conditions. For example, if a window was assigned a topology ((B,C),A) (consistent with introgression between B and C) but this window contains two loci with different introgression histories—one with B->C introgression and one with no introgression—then this window will return a measure of AC divergence that is less likely to reject the null hypothesis (C -> B) in favor of the ‘true’ direction (B -> C). This is because the heterogeneous introgression histories of the two loci within this window tend to reduce the true signal of introgression direction (in this case, reduced AC divergence). In other words, when there is substantial heterogeneity in the introgression history between loci within each window, and the true direction of introgression differs from the null hypothesis, this test will tend to be conservative with respect to rejecting the null hypothesis. Interestingly, in our data we find that we reject the null hypothesis (that is, we infer B->C as the direction of introgression) relatively frequently (see Results), suggesting that any effect of within-window recombination has not substantially reduced our ability to detect deviations from the null within our dataset.

*Defining and determining geographical proximity, mating system difference, and evolutionary distance*

To test if spatial proximity increases the incidence of hybridization, we defined geographically closer lineages (P2 and P3) and geographically distant lineages (P1 and P3) based on known geographical ranges for our species and physical collection locations of the sequenced accessions. For these analyses, we only included species pairs that are known to be geographically proximate within their native ranges. To identify these species pairs, we used the collection location data (downloaded from tgrc.ucdavis.edu) to determine the geographical location of all accession records for each species, and then identified the subset for which the geographic distance between at least one accession of each species pairing was less than 5 km for our geographically closer lineages. Five km was chosen as a reasonable maximum cutoff for defining geographical proximity because a number of ecological studies, including in *Solanum*, suggest it is within potential dispersal distances for bee pollinators (Rick, 1950). Within this set of species pairs, we then identified appropriate accessions from the 32 sequenced accessions to use as P1, P2, and P3 in our 4-taxon tests excluding *S. tuberosom* (outgroup)

For P2 and P3, we identified the geographically closest accessions for two species, based on inferred pairwise geographic distances among all sequenced accessions. In addition, we required that the P2 accession must be located within the known range of geographical overlap with the P3 species. To define a geographically distant (P1) accession to contrast with P2 from the same species, we chose the accession with the largest distance from the focal P3 accession, with a minimum geographical distance of at least 100km, from among our 32 sequenced accessions. Note that this P1 lineage could occur within the geographical range of overlap with the second species, but the specific P1 and P3 accessions must be greater than 100 km from each other.

From these criteria, 14 different comparisons (4-taxon tests) could be made (Table 1 and Supplemental Table 2). Because the actual geographic distances among these available accessions varies widely, this analysis is an imperfect reflection of close spatial proximity (sympatry); however, the structure of the tests means we are still systematically polarizing our comparisons to assess the effect of more (closer) versus less (distant) geographic proximity. We also directly considered quantitative differences in geographical proximity between P1 and P2 with accessions from the P3 species in our analyses.

For mating system tests, in our dataset there are three Solanum species for which we had whole genome sequence data for at least one SI and SC accession (*S. arcanum*, *S. habrochaites*, or *S. peruvianum*), allowing us to test if introgression with a second species is more likely given mating system similarities in five different trio combinations (Table 1). For each 4-taxon test, the P1 and P2 position are occupied by conspecific self-incompatible and self-compatible accessions, while the P3 position is an accession from different species that has a mating system that matches the P2 mating system (Figure 1). For example, where P1 was self-incompatible (SI) and P2 was SC, P3 was an SC accession of a different species. With this structure, we also expect a systematic excess of positive *D*-statistics. For combinations that involve *S. pimpinellifolium* in the P3 position (the three trios used exclusively for mating system tests; Table 1), we also assessed whether the specific accession identity (*S. pimpinefollium* LA0373 or LA0400) affected our tests, but found it did not. That is, replicate trios with accession LA0400 in the P3 position (labelled v2 in Table S2, S3, S6, and S7) gave the same results as the trios in which accession LA0373 was used in the P3 position (arcSI.arcSI.pimSCv2: meanD=0.035, sdD=0.012, 95CI=0.011-0.06, P=0.001; habSI.habSC.pimSCv2: meanD=0.013, sdD=0.012, 95CI=-0.011-0.037, P=0.142; perSI.perSC.pimSCv2: meanD=0.024, sdD=0.008, 95CI=0.008-0.041, P<0.001) and inferred the same direction of introgression. Note also that although we did not use geographical criteria to select our mating system trios, all of the P1/P2 accessions used specifically in the mating system comparisons are in close physical proximity to a P3 accession (Table S4), certainly within the range of geographical proximity observed in the ‘proximate’ comparisons that were used for the tests of ‘geography’. Therefore, we’re confident that we are evaluating evidence for introgression in cases where it is, in principle, geographically/biologically possible.

To evaluate whether D statistics change systematically over increasing evolutionary divergence, we estimated overall genetic divergence between species in each trio within each 4-taxon test by averaging the two estimates of pairwise genetic distance between P1 and P3 and between P2 and P3. Genetic distance was calculated as the total number of sites that differed divided by total number of sites for each pairwise comparison using MVFtools CalcPairwiseDistances. Our estimates of genetic distance ranged from 0.01 to 0.17 (see Results, Supplementary Table 6) which is within the range expected for pairwise genome-wide divergence in this group (Aflitos et al., 2014; Lin et al., 2014; Pease et al., 2016). We then assessed the relationship between our estimate of mean genetic distance and the mean genome-wide estimate of D, across our assessed trios.

*Examining the association between introgression patterns and loci known to be associated with interspecific pollen-pistil barriers*

One expectation of mating system comparisons is that introgression patterns along the genome could be influenced by the genomic location of loci known to affect mating-system-related crossing compatibility. In the Solanaceae, several chromosomal regions have previously been associated both with the expression of gametophytic self-incompatibility and with pollen-pistil barriers between species (in particular, pistil-side barriers in which SI species reject pollen from SC species or ‘unilateral incompatibility’ (UI)) (Bernacchi and Tanksley, 1997; Li and Chetelat, 2014). In wild *Solanum*, these include loci on chromosome 1 (the S-locus itself), 12 (likely HT protein; Tovar-Méndez et al., 2017) and 3 (an unidentified gene(s) that magnifies the expression of pistil-side interspecific pollen rejection; Hamlin et al., 2017). Therefore, we further examined whether patterns of introgression along chromosomes 1, 3, and 12 in our mating system analysis were associated with these regions. To do so, we generated a list of genes located in chromosomal blocks that showed highly significant D-statistics in our analyses (Supplementary Figure 4). First, we identified the genomic coordinates along each chromosome at which there was a shift to a block in which there was overrepresentation of the alternative (minority) topology (i.e. higher counts in ABBA or BABA vs. BBAA); we considered a ‘block’ to be a location in which there were at least ten consecutive 100 kb windows which demonstrated an overrepresentation of either ABBA or BABA sites. Using these genomic coordinates, *Bedtools* (Quinlan and Hall, 2010), and the *ITAG2.4* gene models, we extracted all known genes located within the introgressed blocks, including genes which were up to 50 kb upstream and downstream from the endpoints of each estimated introgressed block (Supplementary Table 8). We found no evidence that chromosomal blocks with highly significant D statistics were associated with loci previously implicated in intra- (SI) and inter-specific (UI) pistil-side mating barriers (Supplemental Table 8a - j).

LITERATURE CITED

Aflitos, S., Schijlen, E., Jong, H. de, Ridder, D. de, Smit, S., Finkers, R., Wang, J., Zhang, G., Li, N., Mao, L., Bakker, F., Dirks, R., Breit, T., Gravendeel, B., Huits, H., Struss, D., Swanson‐Wagner, R., Leeuwen, H. van, Ham, R.C.H.J. van, Fito, L., Guignier, L., Sevilla, M., Ellul, P., Ganko, E., Kapur, A., Reclus, E., Geus, B. de, Geest, H. van de, Hekkert, B. te L., Haarst, J. van, Smits, L., Koops, A., Sanchez‐Perez, G., Heusden, A.W. van, Visser, R., Quan, Z., Min, J., Liao, L., Wang, X., Wang, G., Yue, Z., Yang, X., Xu, N., Schranz, E., Smets, E., Vos, R., Rauwerda, J., Ursem, R., Schuit, C., Kerns, M., Berg, J. van den, Vriezen, W., Janssen, A., Datema, E., Jahrman, T., Moquet, F., Bonnet, J., Peters, S., 2014. Exploring genetic variation in the tomato (Solanum section Lycopersicon) clade by whole-genome sequencing. Plant J. 80, 136–148. https://doi.org/10.1111/tpj.12616

Bernacchi, D., Tanksley, S.D., 1997. A n interspecific backcross of Lycopersicon esculentum x L. hirsutum: linkage analysis and a QTL study of sexual compatibility factors and floral traits. Genetics 147, 861–877.

Bolger, A.M., Lohse, M., Usadel, B., 2014. Trimmomatic: a flexible trimmer for Illumina sequence data. Bioinforma. Oxf. Engl. 30, 2114–2120. https://doi.org/10.1093/bioinformatics/btu170

Hamlin, J.A.P., Sherman, N.A., Moyle, L.C., 2017. Two Loci Contribute Epistastically to Heterospecific Pollen Rejection, a Postmating Isolating Barrier Between Species. G3 GenesGenomesGenetics 7, 2151–2159. https://doi.org/10.1534/g3.117.041673

Li, H., 2013. Aligning sequence reads, clone sequences and assembly contigs with BWA-MEM. ArXiv13033997 Q-Bio.

Li, H., Handsaker, B., Wysoker, A., Fennell, T., Ruan, J., Homer, N., Marth, G., Abecasis, G., Durbin, R., 1000 Genome Project Data Processing Subgroup, 2009. The Sequence Alignment/Map format and SAMtools. Bioinforma. Oxf. Engl. 25, 2078–2079. https://doi.org/10.1093/bioinformatics/btp352

Li, W., Chetelat, R.T., 2014. The Role of a Pollen-Expressed Cullin1 Protein in Gametophytic Self-Incompatibility in Solanum. Genetics 196, 439–442. https://doi.org/10.1534/genetics.113.158279

Lin, T., Zhu, G., Zhang, J., Xu, X., Yu, Q., Zheng, Z., Zhang, Z., Lun, Y., Li, S., Wang, X., Huang, Z., Li, Junming, Zhang, C., Wang, T., Zhang, Yuyang, Wang, A., Zhang, Yancong, Lin, K., Li, C., Xiong, G., Xue, Y., Mazzucato, A., Causse, M., Fei, Z., Giovannoni, J.J., Chetelat, R.T., Zamir, D., Städler, T., Li, Jingfu, Ye, Z., Du, Y., Huang, S., 2014. Genomic analyses provide insights into the history of tomato breeding. Nat. Genet. 46, 1220–1226. https://doi.org/10.1038/ng.3117

Pease, J.B., Haak, D.C., Hahn, M.W., Moyle, L.C., 2016. Phylogenomics Reveals Three Sources of Adaptive Variation during a Rapid Radiation. PLoS Biol. 14, e1002379. https://doi.org/10.1371/journal.pbio.1002379

Pease, J.B., Rosenzweig, B.K., 2018. Encoding Data Using Biological Principles: The Multisample Variant Format for Phylogenomics and Population Genomics. IEEE/ACM Trans. Comput. Biol. Bioinform. 15, 1231–1238. https://doi.org/10.1109/TCBB.2015.2509997

Quinlan, A.R., Hall, I.M., 2010. BEDTools: a flexible suite of utilities for comparing genomic features. Bioinforma. Oxf. Engl. 26, 841–842. https://doi.org/10.1093/bioinformatics/btq033

Rick, C.M., 1950. Pollination Relations of Lycopersicon Esculentum in Native and Foreign Regions. Evolution 4, 110–122. https://doi.org/10.1111/j.1558-5646.1950.tb00046.x

Roselius, K., Stephan, W., Städler, T., 2005. The relationship of nucleotide polymorphism, recombination rate and selection in wild tomato species. Genetics 171 753 - 763. https://doi.org/10.1534/genetics.105.043877

The Tomato Genome Consortium, 2012. The tomato genome sequence provides insights into fleshy fruit evolution. Nature 485, 635–641. https://doi.org/10.1038/nature11119

Tovar-Méndez, A., Lu, L., McClure, B., 2017. HT proteins contribute to S-RNase-independent pollen rejection in Solanum. Plant J. Cell Mol. Biol. 89, 718–729. https://doi.org/10.1111/tpj.13416
